# Arf6 controls retromer traffic and intracellular cholesterol distribution via a phosphoinositide-based mechanism

**DOI:** 10.1038/ncomms11919

**Published:** 2016-06-23

**Authors:** Catherine Marquer, Huasong Tian, Julie Yi, Jayson Bastien, Claudia Dall'Armi, YoungJoo Yang-Klingler, Bowen Zhou, Robin Barry Chan, Gilbert Di Paolo

**Affiliations:** 1 Department of Pathology and Cell Biology, Columbia University Medical Center, New York City, New York 10032, USA

## Abstract

Small GTPases play a critical role in membrane traffic. Among them, Arf6 mediates transport to and from the plasma membrane, as well as phosphoinositide signalling and cholesterol homeostasis. Here we delineate the molecular basis for the link between Arf6 and cholesterol homeostasis using an inducible knockout (KO) model of mouse embryonic fibroblasts (MEFs). We find that accumulation of free cholesterol in the late endosomes/lysosomes of *Arf6* KO MEFs results from mistrafficking of Niemann–Pick type C protein NPC2, a cargo of the cation-independent mannose-6-phosphate receptor (CI-M6PR). This is caused by a selective increase in an endosomal pool of phosphatidylinositol-4-phosphate (PI4P) and a perturbation of retromer, which controls the retrograde transport of CI-M6PR via sorting nexins, including the PI4P effector SNX6. Finally, reducing PI4P levels in KO MEFs through independent mechanisms rescues aberrant retromer tubulation and cholesterol mistrafficking. Our study highlights a phosphoinositide-based mechanism for control of cholesterol distribution via retromer.

## Abstract (web summary)

The small GTPase Arf6 regulates intracellular transport, phosphoinositide signalling and cholesterol homeostasis. Here, Marquer *et al*. show that loss of Arf6 causes cholesterol accumulation in endosomes due to defects in phosphoinositide-dependent retromer-mediated trafficking of CI-M6PR and NPC2.

## Graphical abstract

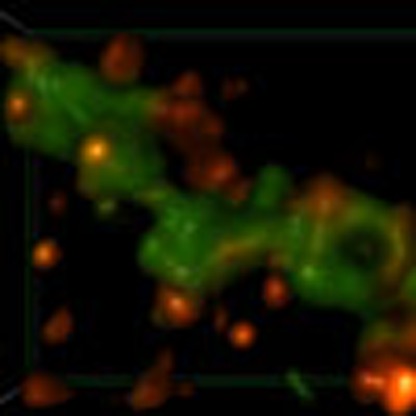

Intracellular transport routes are under strict regulatory control in eukaryotic cells to ensure proper sorting of cargoes, maintain organelle identity and ultimately guarantee cell homeostasis. Among the key regulators of intracellular trafficking pathways, small GTPases, such as ADP ribosylation factor (Arf) family members, play a fundamental role in a compartment-specific manner. Similar to other GTPases, Arf proteins cycle between an inactive GDP-bound form and an active GTP-bound form^1^. Unlike the other Arf family members (that is, Arf1-5), Arf6 is uniquely localized to the plasma membrane and to endosomes^2^,^3^, where it influences membrane trafficking. The role of Arf6 in various clathrin-dependent and -independent endocytic pathways as well as in recycling to the plasma membrane has been extensively studied^4^,^5^. A role for Arf6 in multivesicular body formation has also been recently described^6^. In addition, Arf6 regulates actin remodeling in such contexts as cell spreading, migration, cytokinesis, phagocytosis and neurite outgrowth^5^,^7^. *In vivo, Arf6* ablation is embryonically lethal in the mouse^8^ but a conditional knockout (KO) model revealed a non-cell autonomous role for neuronal Arf6 in oligodendrocyte precursor cell migration and myelination^9^.

One of the major mechanisms of action of Arf6 occurs through the control of lipid metabolism. Indeed, Arf6 binds and activates phosphatidylinositol-4-phosphate 5-kinases (PI4P5Ks), also known as type I PIPKs (PIPKIs), which phosphorylate PI4P into PI(4,5)P_2_ (ref. 10, 11). In addition, Arf6 can activate phospholipase D (PLD)^12^, whose product phosphatidic acid can in turn activate PIPKIs (ref. 13). Overexpressing a constitutively active mutant of Arf6 (Arf6 Q67L) also results in enlarged endosomes that contain high levels of PI(4,5)P_2_ (ref. 14). More recently, Arf6 has been implicated in the regulation of cellular cholesterol distribution. In cultured cells, most cholesterol is derived from cholesteryl ester-rich LDL particles present in the media. LDL-particles are internalized by the LDL receptor (LDLR) and trafficked to the lumen of late endosomes/lysosomes (LE/LYS). Cholesteryl esters are first hydrolysed by lysosomal acid lipase to free cholesterol, which is believed to be transferred by NPC2, a small soluble protein of the LE/LYS lumen, to the polytopic membrane protein NPC1. The latter mediates the egress of cholesterol from the endolysosomal system, allowing for its distribution to other cellular compartments and inhibition of cholesterol biosynthesis via the cholesterol-sensing machinery operating in the endoplasmic reticulum^15^,^16^. Perturbation of cholesterol traffic through mutations of *NPC1* or *NPC2* genes causes Niemann–Pick Type C (NPC) disease, a fatal neurodegenerative disorder associated with accumulation of free cholesterol and other lipids in the endolysosomal system^17^. Several studies provided hints that Arf6 is involved in the control of cholesterol homeostasis. First, Arf6 Q67L enlarged endosomes contain high levels of free cholesterol, labelled with filipin^18^. Second, silencing *Arf6* was found to increase cellular cholesterol content in HeLa cells, reminiscent of an NPC disease phenotype^19^. In addition, *Arf6* and components of its interactome were identified as ‘hits' in a transcriptomic screen performed after cholesterol levels of cultured rat neurons were acutely increased^20^. More recently, *Arf6* was a hit in a genome-wide RNA-interference screen designed to identify genes required for intracellular transport of LDL-derived cholesterol, although this link was not explored further^21^. While these studies point to a link between Arf6 and cholesterol metabolism, the molecular basis underlying this relationship is unknown.

To address this fundamental question, we developed a novel model of *Arf6* conditional KO in immortalized mouse embryonic fibroblasts (MEFs). We found that Arf6 depletion leads to cholesterol redistribution and accumulation in LE/LYS, a phenotype due to the mistargeting of NPC2 away from lysosomes. Because NPC2 is a cargo of the mannose-6-phosphate receptor (M6PR)^22^, we examined the function of retromer, which regulates the retrograde transport of the M6PR. We found that retromer function is impaired in *Arf6* KO cells, thus leading to mislocalization of the cation-independent M6PR (CI)-M6PR away from the *trans*-Golgi network (TGN). We also found that PI4P levels were increased in *Arf6* KO MEFs and that retromer-positive aberrant tubules emerged in part from PI4P-containing endosomes. Finally, we demonstrated that restoring PI4P levels rescues aberrant retromer tubules and cholesterol accumulation. This work therefore highlights a novel role for Arf6 in the regulation of retromer with critical implications for PI4P and cholesterol metabolism.

## Results

### Cholesterol redistributes to LE/LYS in *Arf6* KO cells

**Figure 1:**
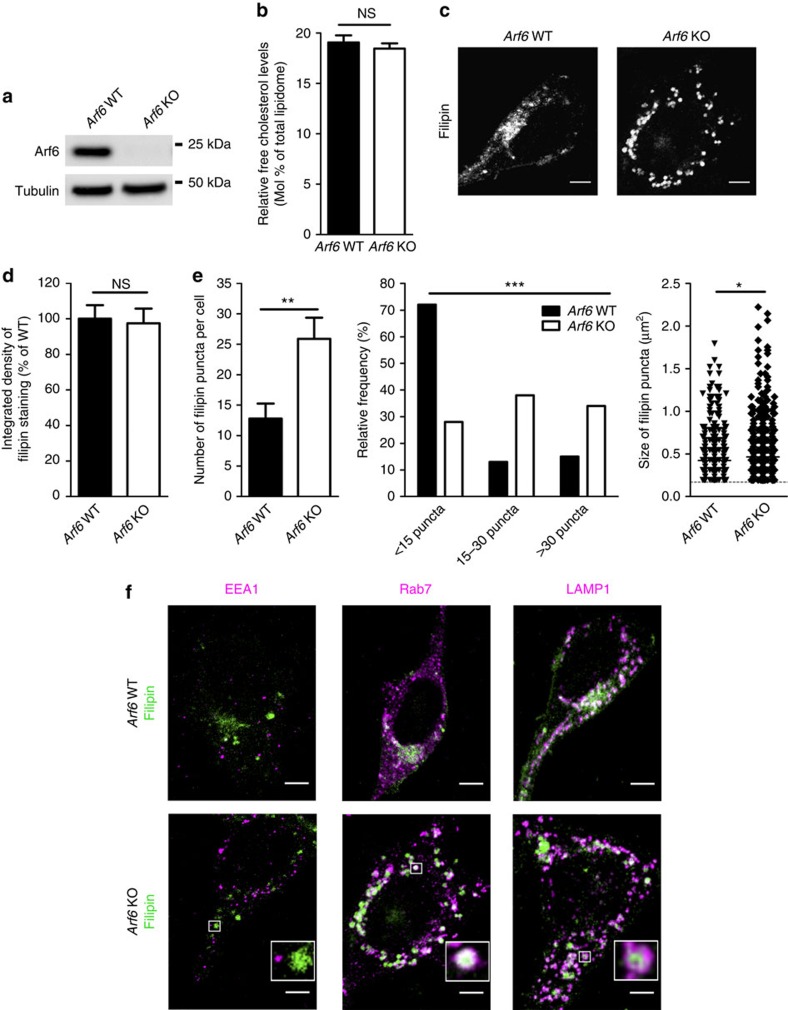
Cholesterol is redistributed to late endosomes/lysosomes in *Arf6* KO MEFs. (**a**) Western blot analysis of endogenous Arf6 levels in Arf6^Flox/Flox^*; Cre-ER* MEFs treated with DMSO (*Arf6* WT) or tamoxifen (*Arf6* KO). Tubulin was used as an equal loading marker. (**b**) Cholesterol levels measured by LC–MS were similar in *Arf6* WT (19.1±0.7 molar % of total lipidome,±indicates s.e.m., *n*=11) and KO cells (18.5±0.5 molar % of total lipidome, *n*=12). NS denotes *P*>0.05 in Student's *t*-test. (**c**) Representative confocal images of *Arf6* WT and KO MEFs stained with filipin. Scale bar, 5 μm. (**d**) Normalized integrated densities of filipin staining were similar in *Arf6* WT (100±8%,±indicates s.e.m., n=32 cells from three experiments) and *Arf6* KO cells (98±8%, *n*=42 cells from four experiments). NS denotes *P*>0.05 in Student's *t*-test. (**e**) Quantification of filipin puncta in *Arf6* WT and KO cells. Left panel, number of puncta per cell in *Arf6* WT (13±2 puncta per cell,±indicates s.e.m., *n*=39 cells from 3 experiments) and *Arf6* KO cells (26±3 puncta per cell, *n*=29 cells from 3 experiments). **P<0.01 in Student's *t*-test. Middle panel, frequency distribution of the number of filipin puncta per cell. ***P<0.001 in *χ*^2^-test. Right panel, scatter dot blot of the size of filipin puncta in *Arf6* WT (0.42±0.01 μm^2^,±indicates s.e.m., 498 puncta from 39 cells) and KO cells (0.47±0.01 μm^2^, 749 puncta from 29 cells). *P<0.05 in Mann–Whitney test. (**f**) Representative immunostainings for EEA1, Rab7 or LAMP1 (magenta) of *Arf6* WT or KO cells labelled with filipin (green). Scale bar, 5 μm. NS, not significant.

To control the ablation of Arf6 acutely and inducibly, MEFs were generated from *Arf6*^Flox/Flox^ embryos also expressing a fusion of Cre recombinase with the ligand-binding domain of the oestrogen receptor (Cre-ER; Supplementary Fig. 1a). After immortalization, MEFs treated with tamoxifen showed gradually reduced levels of Arf6 protein by western blotting, leading to a complete absence of protein after 6 days (Fig. 1a; Supplementary Fig. 1b). We then assessed the impact of Arf6 ablation on cholesterol. Cholesterol content was first analysed by liquid chromatography–mass spectrometry (LC–MS) and was similar in *Arf6* WT and KO MEFs (Fig. 1b). MEFs were subsequently stained with filipin, a fluorescent probe for free cholesterol (Fig. 1c). The overall filipin intensity was comparable in the two genotypes (Fig. 1d), in agreement with the LC–MS results. However, while wild-type (WT) cells exhibited a diffuse staining at the plasma membrane and the cytoplasm with higher fluorescence intensity in the perinuclear region, the pattern of filipin staining was altered in KO MEFs, where it accumulated in cytoplasmic puncta (Fig. 1c). Quantification of filipin fluorescence revealed there were more cholesterol puncta in *Arf6* KO cells compared with controls (Fig. 1e). Filipin puncta in *Arf6* KO MEFs also appeared significantly larger than in the controls (Fig. 1e). Aberrant filipin distribution could be rescued by expressing ARF6-HA (ref. 3) in *Arf6* KO cells (Supplementary Fig. 2), indicating that the cholesterol redistribution phenotype is a direct and specific consequence of Arf6 ablation in those cells. We then identified the specific compartment where free cholesterol accumulated in *Arf6* KO cells. Filipin accumulated in Rab7- and LAMP1-positive organelles but not in EEA1-positive organelles (Fig. 1f), indicating that cholesterol is redistributed to LE/LYS but not to early endosomes in KO cells. LAMP1 protein levels (Supplementary Fig. 3a) as well as the number and size of LAMP1-positive organelles (Supplementary Fig. 3b) were similar in both genotypes, indicating that Arf6 depletion did not cause any major structural disruption of LE/LYS.

### NPC2 mistrafficking causes cholesterol redistribution

**Figure 2:**
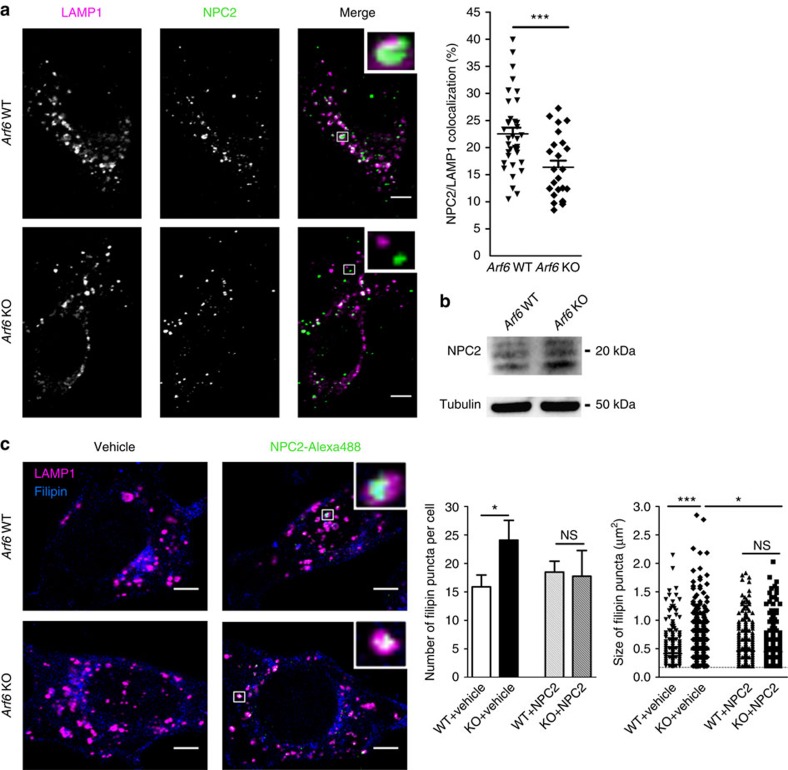
NPC2 mistrafficking causes cholesterol redistribution in *Arf6* KO MEFs. (**a**) Left panel, representative confocal images of *Arf6* WT and KO cells immunostained for endogenous LAMP1 (magenta) and NPC2 (green). Scale bar, 5 μm. Right panel, NPC2/LAMP1 co-localization levels in *Arf6* WT (23±1%,±indicates s.e.m., *n*=36 cells from three experiments) and *Arf6* KO cells (16±1%, *n*=24 cells from three experiments). ***P<0.001 in Student's *t*-test. (**b**) Western blot analysis of endogenous NPC2 levels in *Arf6* WT and KO cells. Tubulin was used as an equal loading marker. NPC2 protein levels were similar (*P*>0.05 in Student's *t*-test) in *Arf6* WT (100±6%,±indicates s.e.m., *n*=3) and KO cells (110±26%, *n*=3). (**c**) NPC2-Alexa488 (green) or vehicle was added to the culture media of *Arf6* WT and KO cells for 24 h. Cells were then washed, fixed and stained for endogenous LAMP1 (magenta) and cholesterol (with filipin, blue). Left panel, representative confocal images. Scale bar, 5 μm. Inset shows that exogenous NPC2 reaches the LAMP1 compartment. Center and right panel, quantification of filipin puncta. Center, number of filipin puncta in vehicle-treated *Arf6* WT (16±2 puncta per cell,±indicates s.e.m., *n*=34 cells, three experiments), vehicle-treated KO (24±3 puncta per cell, *n*=35 cells, three experiments), NPC2-treated *Arf6* WT (18±2 puncta per cell, *n*=36 cells, three experiments) and NPC2-treated KO cells (18±5 puncta per cell, *n*=28 cells, three experiments). NS and * stand for *P*>0.05 and *P*<0.05, respectively, in *t*-test with Welch's correction. Right, scatter dot blot of the size of filipin puncta of vehicle-treated *Arf6* WT (0.41±0.01 μm^2^,±indicates s.e.m., 540 puncta from 34 cells), vehicle-treated *Arf6* KO (0.48±0.01 μm^2^, 843 puncta from 35 cells), NPC2-treated *Arf6* WT (0.46±0.01 μm^2^, 663 puncta from 36 cells) and NPC2-treated *Arf6* KO cells (0.45±0.01 μm^2^, 496 puncta from 28 cells). NS denote *P*>0.05, *P<0.05 and ***P<0.001 in Mann–Whitney test, respectively. NS, not significant.

Since the accumulation of cholesterol in LE/LYS is reminiscent of NPC cells^17^, we assessed whether the localization of NPC1 and/or NPC2 is affected by Arf6 depletion. As probed by Western blot, NPC1 protein levels were comparable (*P*>0.05, Student's *t*-test) in *Arf6* WT (100±9%,±indicates s.e.m., *n*=3) and *Arf6* KO cells (75±20%, *n*=3; Supplementary Fig. 3c). NPC1-GFP was found on the membrane of LE/LYS in both *Arf6* WT and KO cells and displayed extensive co-localization with LAMP1, independently of Arf6 presence (Supplementary Fig. 3d), suggesting that it is correctly trafficked in *Arf6* KO cells. In contrast, co-localization of endogenous NPC2 and LAMP1 was greatly reduced in *Arf6* KO cells compared with controls (Fig. 2a), although NPC2 protein levels were comparable in both genotypes (Fig. 2b; *Arf6* WT 100±6% and *Arf6* KO cells 110±26%,±indicates s.e.m., *n*=3). The reduced presence of NPC2 in the LE/LYS compartment coincided with greater co-localization of this protein with Golgin97, a TGN marker (Supplementary Fig. 4), leading to the hypothesis that mistargeting of NPC2 and its thus reduced activity may be responsible for cholesterol accumulation in LE/LYS. To test this, we bypassed the anterograde pathway for delivery of NPC2 to endosomes by exogenously adding fluorescent NPC2-Alexa488 to MEFs and monitored cholesterol distribution with filipin (Fig. 2c). In vehicle-treated KO cells, cholesterol accumulated in LE/LYS as expected (Fig. 2c). Exogenous NPC2 was able to be internalized and reach the LAMP1-positive LE/LYS compartment and, remarkably, decreased both the number and size of filipin puncta in the *Arf6* KO cells (Fig. 2c), corroborating our hypothesis that Arf6 depletion induces cholesterol redistribution to LE/LYS through NPC2 mistrafficking.

### Mislocalisation of CI-M6PR causes mistrafficking of NPC2

**Figure 3:**
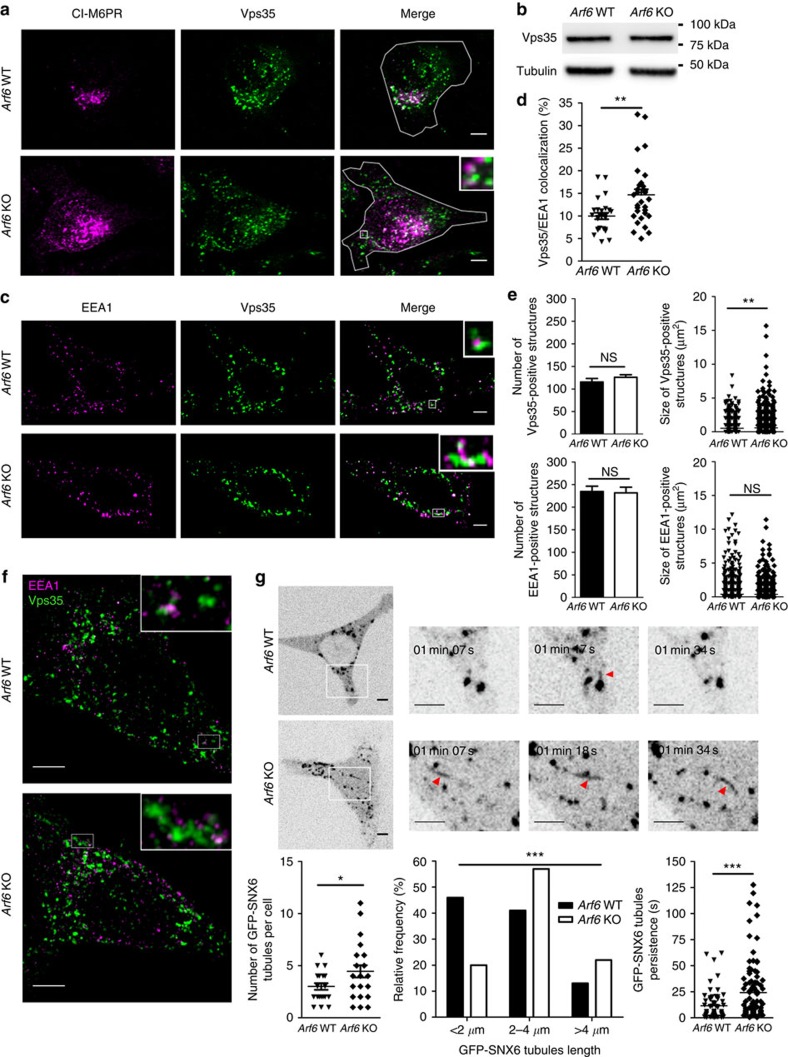
Retromer function is impaired in *Arf6* KO MEFs. (**a**) Representative maximum intensity projections of *Arf6* WT and KO cells immunostained for CI-M6PR (magenta) and Vps35 (green). Scale bar, 5 μm. (**b**) Western blot analysis of Vps35 levels in *Arf6* WT (100±5%, *n*=5) and KO cells (113±5%, *n*=5), *P*>0.05 in Student's *t*-test. Tubulin was used as an equal loading marker. (**c**) Representative confocal images of *Arf6* WT and KO cells immunostained for EEA1 (magenta) and Vps35 (green). Scale bar, 5 μm. (**d**) Levels of Vps35/EEA1 co-localization in *Arf6* WT (10±1%, *n*=26 cells) and KO cells (15±1%, *n*=30 cells). **P<0.01 in *t*-test with Welch's correction. (**e**) Top left panel, number of Vps35 puncta in *Arf6* WT (116±8 puncta per cell, *n*=28 cells) and KO cells (126±6 puncta per cell, *n*=28 cells). ns stands for *P*>0.05 in Student's *t*-test. Top right, size of Vps35 puncta in WT (0.53±0.01 μm^2^, 3,241 puncta) and KO cells (0.59±0.01 μm^2^, 3,529 puncta). ** stands for *P*<0.01 in Mann–Whitney test. Bottom left, number of EEA1 puncta in WT (235±12 puncta per cell, *n*=28 cells) and KO cells (232±13 puncta per cell, *n*=23 cells). Bottom right, size of EEA1 puncta in WT (0.40±0.01 μm^2^, 6,571 puncta) and KO cells (0.39±0.01 μm^2^, 5,334 puncta). ns stands for *P*>0.05 in Student's *t*-test (left) and Mann–Whitney test (right). (**f**) Maximum intensity projections of 3D SIM z-stacks of *Arf6* WT and KO cells immunostained for EEA1 (magenta) and Vps35 (green). Scale bar, 5 μm. (**g**) Spinning-disk confocal imaging of *Arf6* WT and KO MEFs expressing GFP-SNX6. Top panel, representative images. Scale bar, 5 and 1 μm (insets). Arrows indicate tubules. Lower panel, quantification of GFP-SNX6 tubules. Left, number of GFP-SNX6 tubules in *Arf6* WT (3±0.3 tubules per cell, *n*=21 cells) and KO cells (4.5±0.6 tubules per cell, *n*=22 cells). *P<0.05 in *t*-test with Welch's correction. Center, frequency distribution of GFP-SNX6 tubules length in *Arf6* WT (2.6±0.2 μm, *n*=63 tubules) and KO cells (3.2±0.2 μm, *n*=97 tubules). ***P<0.001 in *χ*^2^-test. Right, persistence of GFP-SNX6 tubules in *Arf6* WT (11.6±1.8 s, *n*=62 tubules) and KO cells (24.1±2.9 s, *n*=97 tubules). ***P<0.001 in *t*-test with Welch's correction. All values are given as mean±s.e.m.

NPC2 trafficking was reported to be dependent on M6PRs, with a more significant contribution of CI-M6PR relative to cation-dependent-M6PR (ref. 22). CI-M6PR cycles between the TGN and endosomes, where it transports its ligands, mostly soluble lysosomal proteins, including hydrolases. The latter are then released in endosomes as a result of a lower pH and passively delivered to the LE/LYS where their function is needed. The retrograde transport of CI-M6PR from endosomes to the TGN is mediated primarily by the retromer complex^23^,^24^. We thus investigated whether NPC2 mistrafficking resulted from deficits in the traffic of CI-M6PR. In *Arf6* WT cells, CI-M6PR was highly concentrated in the perinuclear region, in accordance with previous reports that it primarily accumulates in the TGN at steady state^25^ (Fig. 3a). However, in *Arf6* KO cells, CI-M6PR appeared more dispersed and was present not only in the TGN area but also on vesicular structures that were positive for Vps35, the core component of retromer (Fig. 3a).

### The retromer complex function is impaired in *Arf6* KO cells

We then asked whether CI-M6PR mislocalization was due to a deficit in its retrograde transport caused by a malfunction of the retromer. The retromer complex is composed of two subcomplexes. The cargo-selective core subcomplex, composed of Vps35, Vps29 and Vps26, is involved in cargo recognition and sorting. The membrane-deforming subcomplex, composed of a sorting nexin (SNX) heterodimer, is involved in tubulo-vesicular carrier formation from endosomal membranes^26^. In the case of CI-M6PR transport, the specific SNX isoforms involved are heterodimers of SNX1 or SNX2 and SNX5 or SNX6 (refs 23, 27, 28, 29, 30).

We thus examined the relationship between Arf6 and retromer using confocal microscopy. Because various antibodies failed to specifically recognize endogenous Arf6 in immunofluorescence applications, two different ARF6 constructs, ARF6-HA (ref. 3) and CyPET-ARF6 (ref. 31), were exogenously expressed in HeLa cells, which were then stained for VPS35. A pool of Arf6 and retromer were found on the same endosomal compartments, suggesting a potential functional interaction (Supplementary Fig. 5). We then assessed the potential impact of Arf6 deletion on retromer levels by focusing on the core subunit of retromer and found that Vps35 protein levels were similar in *Arf6* WT and KO cells (Fig. 3b). However, the co-localization between Vps35 and EEA1, an early endosome marker, was increased in *Arf6* KO cells, compared with WT cells (15% versus 10%, respectively, Fig. 3c,d), showing that retromer was partially redistributed to early endosomes. While the number of tubulo-vesicular Vps35-positive retromer carriers was comparable, their size was increased in *Arf6* KO cells (Fig. 3e). In contrast, the number and size of EEA1-positive early endosomes were similar in both genotypes (Fig. 3e). When visualized with super-resolution three-dimensional (3D)-Structured Illumination Microscopy (SIM), the size of retromer tubulo-vesicular carriers appeared larger in *Arf6* KO cells, where Vps35 and EEA1 formed a continuous alveolar-shaped endosomal network (Fig. 3f; Supplementary Movies 1, 2, 3, 4).

To test for potential changes in the dynamics of retromer tubules, we transfected MEFs with GFP-SNX6 and imaged them by spinning-disk microscopy (Supplementary Movies 5 and 6). As shown in Fig. 3g, there were more GFP-SNX6-positive tubules in *Arf6* KO cells than in controls, and these tubules were both longer and more persistent. SNX6 tubules were still able to move towards the perinuclear region in *Arf6* KO cells (Supplementary Movies 6), indicating no gross malfunction of molecular motors/microtubules transport. Altogether, our data suggest that Arf6 ablation causes alterations in retromer tubule dynamics, which are consistent with fission defects.

### Retromer knockdown causes cholesterol increase in LE/LYS

**Figure 4:**
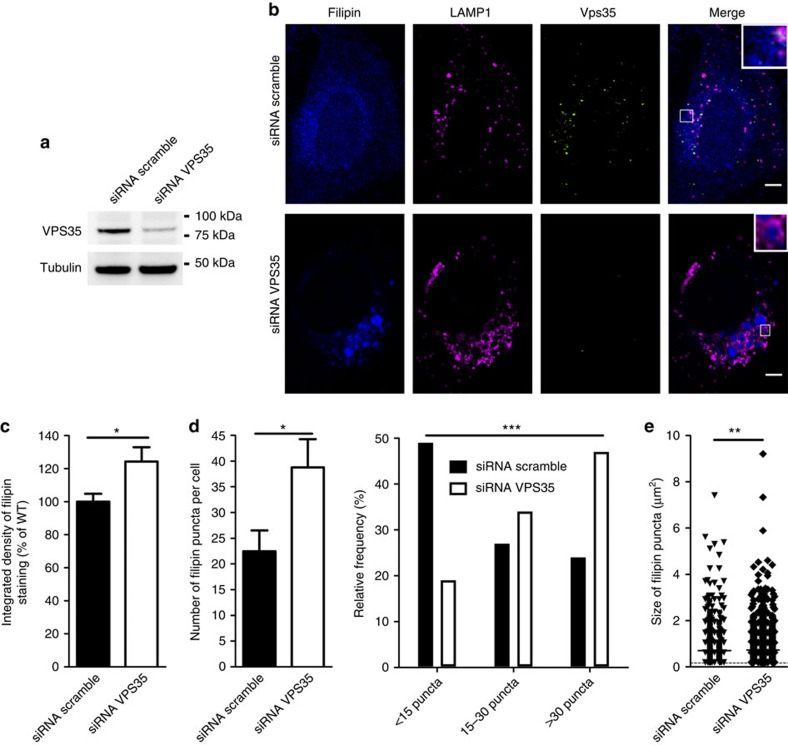
Cholesterol is redistributed to late endosomes/lysosomes in VPS35 KD HeLa cells. (**a**) Western blot analysis of VPS35 levels in HeLa cells transfected with scramble or VPS35 siRNA. Tubulin was used as an equal loading marker. (**b**) Representative confocal images of HeLa cells transfected with scramble or VPS35 siRNA, immunostained for LAMP1 (magenta) and VPS35 (green) and labelled with filipin (blue). Scale bar, 5 μm. (**c**) Normalized integrated densities of filipin staining in scramble (100±5%,±indicates s.e.m., *n*=29 cells, three experiments) and VPS35 siRNA cells (124±9%, *n*=28 cells, three experiments). *P<0.05 in *t*-test with Welch's correction. (**d**) Quantification (left) and frequency distribution (right) of the number of filipin puncta in scramble (22±4 puncta per cell, ± indicates s.e.m., *n*=33 cells, three experiments) and VPS35 siRNA cells (39±5 puncta per cell, *n*=32 cells, three experiments). *P<0.05 in Student's *t*-test and ***P<0.001 in χ^2^-test. (**e**) Scatter dot blot of the size of filipin puncta in scramble (0.71±0.03 μm^2^, ± indicates s.e.m., 741 puncta from 33 cells) and VPS35 siRNA cells (0.73±0.02 μm^2^, 1,241 puncta from 32 cells). **P<0.01 in Mann–Whitney test.

Our data suggest that Arf6 deletion causes a retromer defect which in turn leads to aberrant localization of CI-M6PR and its cargo NPC2 and finally to accumulation of cholesterol in LE/LYS. If this hypothesis is correct, then retromer deficiency should mimic the cholesterol phenotype observed in *Arf6* KO cells. We thus treated HeLa cells with small interfering (siRNAs) to VPS35 and monitored the cholesterol distribution. After VPS35 siRNA transfection (VPS35 KD), VPS35 protein levels were decreased to 22±11% (± indicates s.e.m., *n*=3) of those found in scramble siRNA-transfected cells, as assessed by western blot (Fig. 4a). Cells treated with siRNAs were fixed, immunostained for LAMP1 and VPS35 and labelled with filipin (Fig. 4b). Unlike *Arf6* KO MEFs, VPS35 KD HeLa cells showed an overall increase in the levels of free cholesterol (Fig. 4c). However, similar to our observations in *Arf6* KO cells, filipin accumulated primarily in large LAMP1-positive puncta (Fig. 4b). Quantification of filipin puncta showed that there was on average ∼77% more puncta/cell in VPS35 KD cells and that there was a significant shift in the relative distribution towards cells with a very large number of puncta (30 or more; Fig. 4d). Filipin puncta were also significantly larger in VPS35 KD cells than in controls (Fig. 4e). An increase in the number of filipin puncta was also observed when HeLa cells were treated with siRNAs targeting SNX1 and SNX2 or SNX5 and SNX6, the main sorting nexin isoforms involved in CI-M6PR trafficking^23^,^27^,^28^,^29^,^30^ (Supplementary Fig. 6). Taken together, these results show that depletion of retromer causes an increase in cholesterol levels and an accumulation of cholesterol in the LE/LYS compartment, consistent with our hypothesis. The retromer KD phenotype appears to be slightly more marked than the *Arf6* KO phenotype vis-à-vis global cholesterol accumulation, perhaps reflecting the impact of a full versus a partial loss of function of retromer in VPS35 KD HeLa cells and *Arf6* KO MEFs, respectively.

### PI4P accumulates on retromer-positive early endosomes

**Figure 5:**
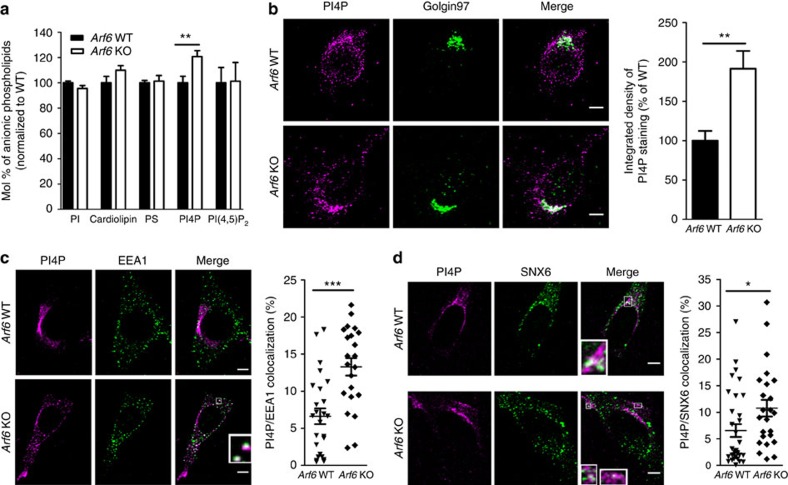
PI4P is increased in *Arf6* KO cells and accumulates in retromer-positive endosomes. (**a**) Bar diagram showing lipid levels in *Arf6* WT and KO cells. Measurements were made by anionic exchange HPLC with suppressed conductivity detection, expressed in molar percentage of total anionic phospholipid measured and normalized to *Arf6* WT levels. PI4P levels were increased in *Arf6* KO cells (121±5%, ± indicates s.e.m., *n*=9) compared with controls (100±5%, *n*=9). **P<0.01 in Student's *t*-test. (**b**) Left, representative confocal images of *Arf6* WT and KO cells immunostained for PI4P (magenta) and Golgin97 (green). Scale bar, 5 μm. Right, normalized integrated densities of PI4P staining were increased in *Arf6* KO cells (191±23%, ± indicates s.e.m., *n*=27 cells, three experiments) compared to controls (100±12%, *n*=22 cells, three experiments). **P<0.01 in *t*-test with Welch's correction. (**c**) Left, representative confocal images of *Arf6* WT and KO cells immunostained for PI4P (magenta) and EEA1 (green). Scale bar, 5 μm. Right, PI4P/EEA1 co-localization levels in *Arf6* WT (7±1%, ± indicates s.e.m., *n*=25 cells, three experiments) and KO cells (13±1%, *n*=22 cells, three experiments). ***P<0.001 in Student's *t*-test. (**d**) Left, representative confocal images of *Arf6* WT and KO cells immunostained for PI4P (magenta) and SNX6 (green). Scale bar, 5 μm. Right, PI4P/SNX6 co-localization levels in WT (7±1%, ± indicates s.e.m., *n*=33 cells, three experiments) and KO cells (11±2%, *n*=24 cells, three experiments). *P<0.05 in Student's *t*-test.

To further delineate the molecular mechanisms underlying the retromer defect in *Arf6* KO cells, we attempted to identify the Arf6 effector responsible for this phenotype. As stated earlier, some of the key effectors of Arf6 are PIPKIs, which catalyse the phosphorylation of PI4P into PI(4,5)P_2_ (refs 10, 11). We thus analysed the anionic phospholipid content of MEFs by HPLC combined with suppressed conductivity. Interestingly, we observed a ∼20% increase in PI4P in *Arf6* KO cells, with no changes in phosphatidylserine, cardiolipin, PI or, more intriguingly, PI(4,5)P_2_ (Fig. 5a). As it was reported that phosphatidic acid levels could also play a role in PIPKIs regulation^13^, we measured phosphatidic acid levels by HPLC and LC–MS. Phosphatidic acid levels measured by HPLC were similar (*P*>0.05, Student's *t*-test) in *Arf6* WT (100±3 normalized molar % of measured lipids,±indicates s.e.m., *n*=3) and *Arf6* KO cells (108±16 normalized molar % of measured lipids, *n*=3). Similarly, phosphatidic acid levels measured by LC–MS were comparable (*P*>0.05, Student's *t*-test) in *Arf6* WT (0.077±0.010 molar % of measured lipids,±indicates s.e.m., *n*=11) and *Arf6* KO cells (0.064±0.007 molar % of measured lipids, *n*=12), making an indirect contribution of PLD unlikely.

Further investigation of PI4P levels by immunofluorescence also revealed a striking approximately twofold increase in PI4P fluorescence in *Arf6* KO MEFs relative to controls (Fig. 5b). As expected^32^, a large pool of PI4P co-localized with the TGN marker Golgin97 in both genotypes (Fig. 5b). However, as recently reported by others^33^, PI4P was also present on more peripheral compartments likely corresponding to endosomes (Fig. 5b). We thus tested whether some of these PI4P-positive compartments are early endosomes by co-immunostaining MEFs for PI4P and EEA1 and found that the co-localization between PI4P and EEA1 was increased by twofold in *Arf6* KO cells compared with controls (Fig. 5c). PI4P thus markedly accumulates on early endosomes. We also assessed whether PI4P accumulates on endosomal pools where retromer is present by immunostaining MEFs for endogenous PI4P and SNX6. We found not only that the overall PI4P/SNX6 co-localization was increased in *Arf6* KO cells compared with WT cells (Fig. 5d, right panel) but also that the cellular region where this co-localization took place was different (Fig. 5d, left panel). While in *Arf6* WT cells, PI4P and SNX6 mostly co-localized in the perinuclear region, they also co-localized in more peripheral compartments in *Arf6* KO cells (Fig. 5d, insets). Comparable results were obtained when immunostaining MEFs for endogenous PI4P and retromer core component Vps35 (Supplementary Fig. 7). We thus concluded that in *Arf6* KO cells, both PI4P and the retromer complex accumulate on endosomes.

### Lowering PI4P levels rescues retromer function

**Figure 6:**
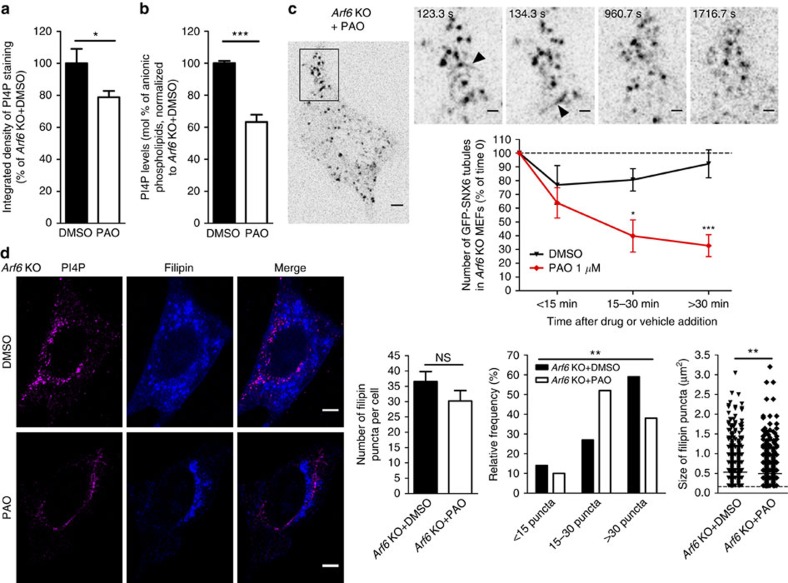
Lowering PI4P levels with PAO rescues excessive retromer tubulation and cholesterol accumulation in LE/LYS in *Arf6* KO cells. (**a**) Normalized integrated densities of PI4P staining (see **d**) of *Arf6* KO cells treated with DMSO (100±9%,±indicates s.e.m., *n*=22 cells, 3 experiments) or PAO (79±4%, *n*=29 cells, three experiments). *P<0.05 in *t*-test with Welch's correction. (**b**) Bar diagram showing PI4P levels in DMSO- and PAO-treated *Arf6* KO cells. Measurements were made by anionic exchange HPLC with suppressed conductivity detection, expressed in molar percentage of measured lipids and normalized to DMSO-treated *Arf6* KO levels. PI4P levels were decreased in PAO-(63±5%, ± indicates s.e.m., *n*=4) compared with DMSO-treated controls (100±1%, *n*=4). ***P<0.001 in Student's *t*-test. (**c**) Kinetic following of GFP-SNX6 tubulation in *Arf6* KO MEFs treated with DMSO or PAO by spinning-disk live imaging. Top panel, representative images of *Arf6* KO cells after different times of PAO treatment. Arrows indicate tubules. Scale bar, 5 and 1 μm (zoom-ins). Lower panel, quantification of the number of GFP-SNX6 tubules after DMSO (black, *n*=9 cells, four experiments) or PAO treatment (red, *n*=9 cells, four experiments), normalized to the number of tubules before the treatment. *P<0.05 and ***P<0.001 in two-way ANOVA with Bonferroni pos*t*-tests, respectively. (**d**) Left, representative immunostaining for PI4P (magenta) of *Arf6* KO cells treated with DMSO or PAO and labelled with filipin (blue). Scale bar, 5 μm. Middle, quantification of the absolute number and relative distribution of filipin puncta in DMSO-(37±3 puncta/cell,±indicates s.e.m., *n*=37 cells, 4 experiments) or PAO-(30±3 puncta per cell, *n*=29 cells, four experiments) treated *Arf6* KO cells. NS denotes *P*>0.05 in Student's *t*-test. **P<0.01 in *χ*^2^-test. Right, size of filipin puncta in DMSO-(0.53±0.01 μm^2^,±indicates s.e.m., 1,352 puncta, 37 cells) or PAO-(0.50±0.01 μm^2^, 876 puncta, 29 cells) treated *Arf6* KO cells. **P<0.01 in Mann–Whitney test. NS, not significant.

These data led us to postulate that endosomal PI4P accumulation may be responsible for the defect in retromer tubules dynamics observed in *Arf6* KO cells (Fig. 3g). To test this, we used phenylarsine oxide (PAO), a PI4K inhibitor^34^, to lower the levels of PI4P. After 30 min of 1 μM PAO treatment, levels of PI4P in *Arf6* KO cells decreased by ∼20% and ∼35%, as assessed by immunostaining and HPLC, respectively (Fig. 6a,b). We quantified the number of GFP-SNX6 tubules in cells treated with dimethyl sulfoxide (DMSO) or PAO and visualized by spinning-disk microscopy (Fig. 6c; Supplementary Movies 7, 9, 9). At time 0, both sets of cells had a similar number of tubules (*P*>0.05, *t*-test with Welch's correction). The number of GFP-SNX6 tubules in DMSO-treated *Arf6* KO MEFs was unchanged with time (*P*>0.05, one-way analysis of variance (ANOVA)). In contrast, PAO-treated *Arf6* KO cells showed a decreased number of tubules after 15 min of treatment (40±12% of the number of tubules at time 0, ± indicates s.e.m.) and this decrease was even more pronounced after 30 min of treatment (33±8% of the number of tubules at time 0). Decreasing the levels of PI4P can thus rescue the defect in retromer tubule dynamics.

### Lowering PI4P levels rescues cholesterol redistribution

**Figure 7:**
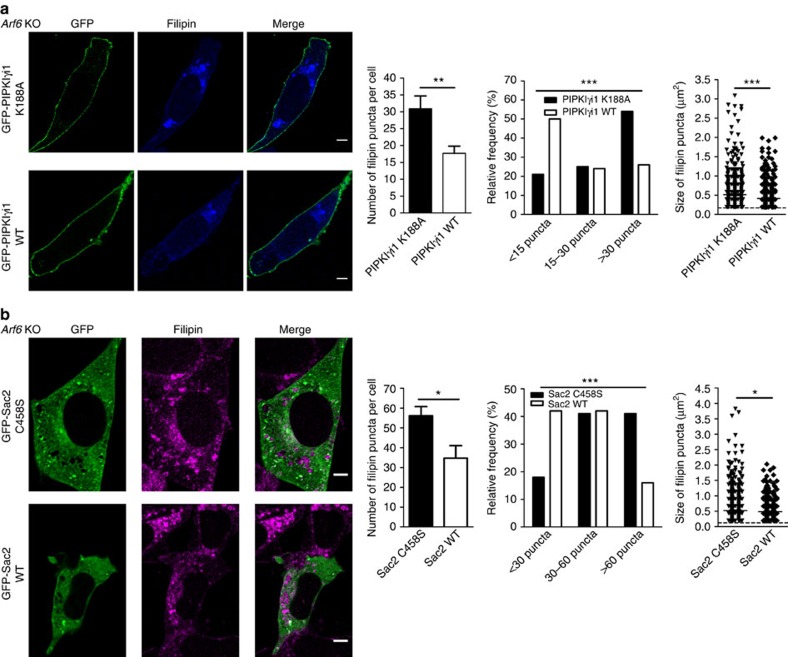
Lowering PI4P levels through GFP-PIPKIγi1 or GFP-Sac2 overexpression rescues cholesterol redistribution in *Arf6* KO cells. (**a**) Left, representative confocal images of *Arf6* KO cells expressing the K188A mutant (top) or the wild-type (bottom) GFP-PIPKIγi1 (green) labelled with filipin (blue). Scale bar, 5 μm. Center, quantification of the absolute number and relative distribution of filipin puncta in *Arf6* KO cells expressing K188A GFP-PIPKIγi1 (31±4 puncta per cell,±indicates s.e.m., *n*=24 cells, three experiments) or WT GFP-PIPKIγi1 (18±2 puncta per cell, *n*=38 cells, five experiments). **P<0.01 in *t*-test with Welch's correction. ***P<0.001 in *χ*^2^-test. Right, size of filipin puncta in *Arf6* KO cells expressing K188A GFP-PIPKIγi1 (0.51±0.01 μm^2^,±indicates s.e.m., 742 puncta, 24 cells) or WT GFP-PIPKIγi1 (0.42±0.01 μm^2^, 671 puncta, 38 cells). ***P<0.001 in Mann–Whitney test. (**b**) Left, representative confocal images of *Arf6* KO cells expressing the C458S mutant (top) or the wild-type (bottom) GFP-Sac2 (green) and labelled with filipin (magenta). Scale bar, 5 μm. Center, quantification of the absolute number and relative distribution of filipin puncta in *Arf6* KO cells expressing C458S GFP-Sac2 (56±5 puncta per cell,±indicates s.e.m., *n*=27 cells, three experiments) or WT GFP-Sac2 (35±6 puncta per cell, *n*=12 cells, three experiments). *P<0.05 in Student's *t*-test. ***P<0.001 in *χ*^2^-test. Right, size of filipin puncta in *Arf6* KO cells expressing C458S GFP-Sac2 (0.52±0.01 μm^2^, ± indicates s.e.m., 1,517 puncta, 27 cells) or WT GFP-Sac2 (0.48±0.02 μm^2^, 417 puncta, 12 cells). *P<0.05 in Mann–Whitney test.

To test whether PI4P increase is also the cause of cholesterol accumulation in the LE/LYS of *Arf6* KO cells, we assessed the impact of lowering PI4P levels on cholesterol localization using different but complementary approaches. First, we used PAO treatment and labelled cells for PI4P and filipin (Fig. 6d). After 30 min of treatment, the average number of filipin puncta per cell was slightly decreased, although it did not reach significance. However, the quantification revealed a shift towards cells with a lower number of puncta in the relative distribution (Fig. 6d, center). The average size of filipin puncta was reduced with a specific decrease in the number of large (1.5-2.5 μm^2^) puncta (Fig. 6d, right). This partial rescue of cholesterol distribution was accompanied by a normalization of CI-M6PR distribution and an increase in NPC2/LAMP1 co-localization after PAO treatment (Supplementary Fig. 8b,c). Next, we overexpressed wild-type GFP-PIPKIγi1 (WT) or its kinase-dead K188A mutant in *Arf6* KO MEFs (Fig. 7a). Quantification of filipin fluorescence in transfected cells after 48 h showed fewer filipin puncta of smaller size in WT-transfected cells (Fig. 7a). The kinase activity of PIPKIγi1 was thus required to rescue the cholesterol redistribution phenotype. To rule out that the changes in cholesterol distribution observed in the *Arf6* KO were not due to the PI(4,5)P_2_ levels increase we surprisingly observed after PAO treatment (Supplementary Fig. 8a), we used a third strategy to selectively decrease PI4P levels in endosomes by exploiting the recently characterized endosomal PI4P-phosphatase Sac2 (refs 35, 36) as a tool. We overexpressed wild-type GFP-Sac2 (WT) or its phosphatase-dead C458S mutant in *Arf6* KO MEFs (Fig. 7b) and found that filipin puncta were fewer and of smaller size in WT GFP-Sac2 transfected cells (Fig. 7b). Cholesterol distribution rescue was thus dependent on the PI4P-phosphatase activity of Sac2. Altogether, these three approaches highlight the role of elevated PI4P levels in cholesterol accumulation in LE/LYS of *Arf6* KO cells.

## Discussion

Arf6 has been implicated in various aspects of cell physiology, primarily as a regulator of vesicular transport to and from the plasma membrane^4^,^5^. A key function of Arf6, however, appears to be the control of lipid signalling and, in particular, phosphoinositide signalling^11^. Parallel studies have also hinted that Arf6 may control cholesterol homeostasis, yet, through unknown mechanisms^18^,^19^,^20^,^21^. This study mechanistically links these disconnected bodies of literature, by identifying a phosphoinositide-based mechanism for the control of intracellular cholesterol distribution via the retromer pathway.

**Figure 8:**
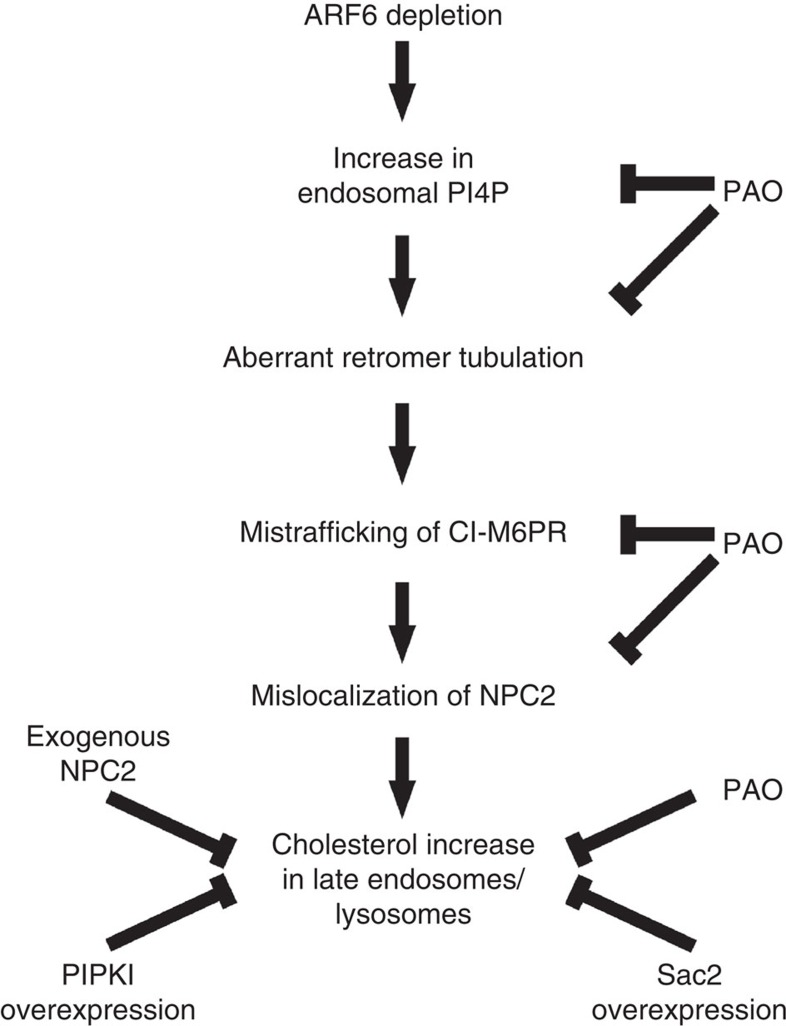
Model of Arf6 regulation of cholesterol homeostasis. Arf6 controls an endosomal pool of PI4P and regulates retromer tubules dynamics in the endosome-to-TGN pathway, consequently impacting CI-M6PR and NPC2 localization (see also text). PAO: PI4K inhibitor.

It is to our knowledge the first time that Arf6 is shown to regulate retromer function, although at least two previous studies presented circumstantial evidence consistent with this idea. First, even before retromer was identified, CI-M6PR was described as being present on Arf6-positive endosomes, but the functional relationship between these two proteins was not addressed^3^. Second, a more recent paper showed that EFA6A, a guanine nucleotide exchange factor for Arf6, interacts with SNX1 and regulates neurite outgrowth through this interaction, although neither the role of retromer nor the link with phosphoinositide signalling and cholesterol homeostasis was addressed in that context^37^. In this study, we propose a novel model whereby Arf6 controls the dynamics of retromer tubules in the endosome-to-TGN pathway responsible for the retrograde transport of CI-M6PR through negative regulation of an endosomal pool of PI4P (Fig. 8). Amongst the potential effectors of PI4P, specific members of the SNX family were considered excellent candidates based on their lipid-binding specificity. In fact, while SNX1, SNX2, SNX5 and SNX6 have all been shown to mediate the retromer-dependent traffic of CI-M6PR transport^23^,^27^,^28^,^29^,^30^ and bind to phosphoinositides via both their PX (phox homology) and BAR (Bin/ amphiphysin/ Rvs) domains, SNX6 appears to be the only member exhibiting a greater affinity for PI4P than for other phosphoinositides^38^, consistent with our findings showing defects in SNX6-coated tubule dynamics in *Arf6* KO cells. Moreover, PI4P has been shown to compete with the p150(Glued) subunit of the dynein/dynactin complex for binding to SNX6 in the TGN^38^. Since impairing the retromer/dynein motor coupling causes defects in retromer tubule fission^39^,^40^, an interesting possibility supported by our data is that the higher levels of PI4P found in the *Arf6* KO cells may enhance the PI4P/SNX6 interaction on endosomal membranes at the expense of the SNX6/p150(Glued) interaction, resulting in retromer fission defects. Whether the lack of Arf6 and its NH_2_-terminal amphipathic helix directly alters membrane tubule dynamics as described for other Arf family members^41^,^42^, and contributes to retromer tubules defects, remains to be investigated. Similarly, a perturbation of the PI4P/PI(4,5)P_2_ ratio on endosomal membranes may alter the fission efficiency of retromer tubules as we have previously reported for endocytic structures^43^.

Our study also further highlights the tight crosstalk between small GTPases and phosphoinositide metabolism^32^,^44^. In the context of retromer regulation, Rab5 has been shown to control class III PI3K (ref. 45), whose product PI3P mediates the recruitment of key components of the retromer assemblies, SNX1 and SNX2 (refs 27, 28, 29, 46). In contrast, Rab7 directly interacts with the VPS core complex to facilitate the recruitment of retromer to endosomal membranes^46^,^47^. In this study, we propose a novel mechanism for GTPase-mediated control of retromer, involving the negative regulation of endosomal PI4P levels. As mentioned earlier, Arf6 controls PIPKIs, suggesting that PI(4,5)P_2_ generation may be key to its biological function. However, the HPLC profile suggests that it is rather the accumulation of PI4P on endosomes than a reduction in PI(4,5)P_2_ which accounts for the retromer phenotype. This is further supported by our rescue experiments showing that decreasing PI4P levels, rather than enhancing PI(4,5)P_2_ levels, corrects the retromer phenotype (Figs 6 and 7). In addition, because PIPKIγ overexpression rescues the cholesterol redistribution phenotype, we speculate that dysregulation of a PIPKI isoform on endosomal membranes is the basis of PI4P accumulation on these compartments. However, we cannot rule out contributions of class II PI3Ks, PI4Ks or PI4P phosphatases to this phenotype, particularly in light of the presence of some of their isoforms in the endosomal compartments, including the Sac2 inositol 4-phosphatase^34^,^35^,^36^,^48^,^49^,^50^,^51^,^52^,^53^,^54^,^55^.

While our study further establishes a link between Arf6 and NPC disease^19^, it also reports a role of retromer in endolysosomal cholesterol homeostasis. As retromer has been linked to several neurodegenerative diseases^56^ and cholesterol accumulation in LE/LYS can lead to neurodegeneration^57^, this raises the question of the importance of the link between retromer and cholesterol in neurodegeneration. It is particularly intriguing that both decreased retromer levels^58^ and increased cholesterol levels^59^,^60^ have been linked to late-onset Alzheimer's disease. Further studies should give us insights into the role of the novel Arf6/retromer/cholesterol connection in the aetiology of neurodegenerative diseases.

## Methods

### Reagents

DMSO, tamoxifen, filipin, phenylarsine oxide (PAO) and bovine serum albumin (BSA) were purchased from Sigma-Aldrich. Saponin was from Acros.

### Antibodies and peptides

The antibody against Arf6 (ref. 61) was a kind gift from Julie Donaldson (NIH, Bethesda, MD; 1/1000 for western blots). The other antibodies were obtained from the following sources: rabbit antibodies to LAMP1 (ab24170, Abcam, 1/500 in immunofluorescence (IF), 1/500 for western blots), Rab7 (9367, Cell Signalling Technology, 1/100 in IF), EEA1 (3288, Cell Signalling Technology, 1/400 in IF), NPC1 (ab36983, Abcam, 1/500 for western blots), NPC2 (sc-33776, Santa Cruz, 1/50 in IF and H00010577-D01P, Abnova, 1/100 for western blots), M6PR (PA3-850, Thermo, 1/100 in IF), Golgin97 (13192, Cell Signalling Technology, 1/100 in IF); goat polyclonal antibodies to SNX1 (ab99286, Abcam, 1/50 in IF), SNX6 (sc-8679, Santa Cruz, 1/50 in IF) and Vps35 (ab10099, Abcam, 1/150 in IF); rat monoclonal antibodies (mABs) to LAMP1 (sc-19992, Santa Cruz, 1/50 in IF) and HA (3F10, Roche, 1/200 in IF); mouse monoclonal antibodies to tubulin (T5168, Sigma, 1/1000 for western blots), NPC2 (sc-166449, Santa Cruz, 1/50 in IF), Vps35 (ab57632, Abcam, 1/300 in IF, 1/1,000 for western blots) and PI4P (Z-P004, Echelon, 1/100 in IF). Purified NPC2-Alexa488 (ref. 62) was a kind gift from Ling Huang and Peter Lobel (Rutgers University, Piscataway, NJ). Peroxidase-conjugated secondary antibodies were from Biorad and were used at a dilution 1/3,000. Alexa-conjugated fluorescent secondary antibodies were from Life Technologies (1/400 for Alexa 488 and Alexa 555; 1/300 for Alexa 647).

### Plasmids and RNA interference

NPC1-GFP was kindly provided by Kara Primmer and Kevin Vaughan (University of Notre Dame, Notre Dame, IN)^63^. GFP-SNX6 was kindly provided by Peter Cullen (University of Bristol, UK)^30^. ARF6-HA^3^ was kindly provided by Julie Donaldson (NIH, Bethesda, MD) and CyPET-ARF6 (ref. 31) was a kind gift from Martin Schwartz (Addgene plasmid # 18840). GFP-Sac2 and its phosphatase-dead mutant GFP-Sac2 C458S were kind gifts from Yuxin Mao (Cornell University, Ithaca, NY)^35^. GFP-PIPKIγi1 and its kinase-dead mutant GFP-PIPKIγi1-K188A were previously published^64^. All DNA sequences were checked by sequencing. VPS35 knockdown (KD) was performed with a 1:1 mix of Qiagen Flexitube siRNA Human VPS35_5 (SI04268131) and Human Vps35_6 (SI04279296). SNX1 and SNX2 double knockdowns were performed with a 1:1 mix of ON-TARGET plus Human SNX1 (6642) siRNA-SMART pool and ON-TARGET plus Human SNX2 (6643) siRNA-SMART pool (GE Dharmacon). SNX5 and SNX6 double knockdowns were performed with a 1:1 mix of ON-TARGET plus Human SNX5 (27131) siRNA-SMART pool and ON-TARGET plus Human SNX6 (58533) siRNA-SMART pool (GE Dharmacon).

### Generation of *Arf6* ^*Flox/Flox*^ mice

To generate *Arf6* conditional KO mice, the entire coding sequence (∼4,000 bp) was flanked with *loxP* sites, with one (*loxP*) sub-cloned into a targeting vector upstream of the *Arf6* coding region at the SspI site and a FRT-NEO-FRT-*loxP* cassette inserted downstream of the *Arf6* sequence at the XhoI site. The targeting vector was electroporated into ES cells and successfully targeted ES cells were microinjected into blastocysts, which were then transferred into foster mothers for the generation of chimeric mice. Resultant *Arf6*^*Flox-Neo/+*^ mice were crossed with Flp Deleter mice (ACTB-Flpe from The Jackson Laboratory, Stock 005703) to generate *Arf6*^*Flox/+*^ mice. These mice were then crossed with the inducible Cre-ER mice (Rosa B6^Cre-ER/Cre-ER^ from the Jackson Laboratory, Stock 008463), to generate *Arf6*^*Flox/+*^*;Cre-ER* mice, which were then interbred to generate *Arf6*^*Flox/Flox*^*;Cre-ER* mice. Animals were used in full compliance with National Institutes of Health/ Institutional Animal Care and Use Committee guidelines. Specifically, adult mice were sacrificed via inhalation of carbon dioxide, followed by cervical dislocation, while embryos derived from the killed mothers were decapitated after inducing hypothermia on ice. The animal protocols were approved by the Committee on the Ethics of Animal Experiments of Columbia University. *Arf6*^*Flox/+*^ mice were made available to The Jackson Laboratory.

### Cell cultures and transfection

Immortalized MEFs and HeLa cells were maintained at 37 °C in a humidified 5% CO_2_ atmosphere in DMEM with Glutamax supplemented with 10% fetal bovine serum and 1% penicillin/streptomycin (all from Life technologies). Cells were negative for mycoplasma contamination. Primary MEFs were generated from embryonic day 13.5 *Arf6*^*Flox/Flox*^*;Cre-ER* mice. Torsos from *Arf6*^*Flox/*Flox^*;Cre-ER* embryos were minced and trypsinized for 15 min and homogenized by pipetting. MEFs were immortalized by multiple passaging and were used for experiments after 25 passages. *Arf6*^*Flox/Flox*^*;Cre-ER* MEFs contain a Cre-oestrogen receptor (Cre-ER) fusion protein, rendering Cre recombinase inducible by tamoxifen^65^. *Arf6*^*Flox/Flox*^*;Cre-ER* MEFs were plated at a high density and treated the next day with DMSO or 10 μM tamoxifen. After 72 h, cells were split in fresh media and left in culture for another 72 h. Arf6 deletion was complete by then. All experiments were performed between 3 and 7 days after complete Arf6 deletion. MEFs were transiently transfected using Lipofectamine LTX (Life Technologies) and protein expression was assessed after 24 or 48 h. HeLa cells were transfected using Lipofectamine 2000 (Life Technologies). Protein expression was assessed after 24 or 48 h and knockdowns were assessed at 72 h.

### Anionic phospholipid analysis

Phosphoinositides and other anionic phospholipids were measured as deacylated lipids using anionic exchange HPLC with suppressed conductivity detection^66^,^67^. Samples were prepared from cells grown to half-confluency in 100-mm dishes, washed with ice-cold HBSS and processed for phosphoinositide enrichment, followed by deacylation^66^,^67^.

### Free cholesterol analysis

Free cholesterol levels (and other lipids) were measured by separating complex lipid mixtures by reverse phase HPLC, followed by detection using multiple reactions monitoring^68^. Samples were prepared from cells grown to half-confluency in 60-mm dishes, washed with ice-cold HBSS and processed for lipid extraction using a modified Bligh and Dyer protocol^68^. Free cholesterol levels were quantified by referencing a D7-cholesterol (Avanti Polar Lipids) internal standard that was spiked into each sample before the analysis.

### Immunofluorescence and filipin staining

For NPC2 uptake experiments, MEFs were exposed to fresh medium containing PBS (Boston BioProducts) or purified NPC2-Alexa488 (ref. 62) 60 nM for 24 h, rinsed twice with HBSS (Life Technologies) and fixed. For PAO experiments, MEFs were exposed to fresh medium containing DMSO or PAO 1 μM for 30 min at 37 °C, rinsed twice with HBSS and fixed. For immunofluorescence experiments, cells grown on glass coverslips were washed once with HBSS and fixed with 4% paraformaldehyde (Electron Microscopy Sciences) for 20 min at room temperature or at 37 °C (when preservation of the tubular structures was crucial). Cells were then washed twice in HBSS and incubated with NH4Cl (50 mM in PBS) for 10 min. For NPC2 staining, cells were fixed for 1 h in Bouin's solution (Sigma-Aldrich). Cells were then washed twice in PBS and permeabilized with solution A (saponin 0.05%, BSA 5% in PBS) for at least 45 min at 37 °C. They were then incubated with primary antibodies diluted in solution A for 1 h at room temperature, washed three times in solution A, incubated with fluorescent secondary antibodies diluted in solution A for 1 h at room temperature and washed again three times in solution A. An extra step was performed for filipin staining. Cells were washed once in PBS, incubated in filipin (0.5 mg ml^−1^ in PBS) for 1 h at room temperature and washed twice with PBS. Cells were finally washed once with PBS and coverslips were mounted in Vectashield mounting medium (Vectorlabs).

### Confocal microscopy

Z-stacks were acquired by confocal laser scanning microscopy (Zeiss LSM 700). Fluorescence was collected with a × 63 plan apochromat immersion oil objective (numerical aperture (NA) 1.4). Extraction of single z-frame and maximum intensity projections were performed with the ImageJ software. Integrated densities, that is, cell area × [(mean cell intensities summed on the whole z-stack)–(mean background intensities summed on the whole z-stack)], were measured with Image J software. Z-stack co-localization percentage, that is, × 100 (Pearson's coefficient)^2^, were obtained with the JACoP plugin of ImageJ^69^. The number and size of puncta were quantified with ICY software^70^. We checked that the cells we were comparing for fluorescence intensity, size and number of organelles or co-localization had comparable areas (*P*>0.05 in Student's *t*-test). Each experiment was independently repeated at least three times, resulting in the analysis of 22 to 42 cells for each condition.

### 3D Structured illumination microscopy

Cells were grown on high performance #1.5 coverslips (Zeiss). Immunofluorescence was performed as described for confocal microscopy but secondary antibodies were used twice as concentrated (1/200) and coverslips were mounted in ProLong Gold (Life technologies). Z-stacks were acquired on a Nikon N-SIM microscope equipped with an iXon Ultra 897 camera (Andor) and a × 100 plan apochromat immersion oil objective (NA 1.49). Image reconstruction and analysis were performed with the NIS-Elements software.

### Spinning-disk confocal live imaging

Cells were grown in 35-mm glass-bottom dishes (MatTek) and imaged 48 h after transfection. Before imaging, medium was replaced to get rid of any debris and to ensure accuracy of volume for the PAO experiments. For kinetic PAO experiments, *Arf6* KO cells expressing GFP-SNX6 were imaged for 2 min and their localization recorded. At time 0, DMSO or PAO (1 μM final) diluted in medium was added to the cells and three movies of 2 min were recorded (before 15 min, between 15 and 30 min and between 30 and 45 min). Only cells displaying at least two tubules at time 0 were kept for analysis. Live imaging was carried out using a Nikon Ti Eclipse microscope equipped with a Yokogawa CSU-X1 spinning-disk, a motorized stage and a Photometrics Evolve 512 camera (Roper Scientific) controlled with the NIS-Elements software. Movies were acquired at 37 °C and 5% CO_2_ with a Tokai Hit stage-top incubator and objective heater. Fluorescence was collected with a 60 × plan apochromat immersion oil objective (NA 1.49). Images were collected at 1 × 1 binning with an exposure time of 100 ms. The number, maximum length and persistence of GFP-SNX6 tubules were analysed with ImageJ software.

### Protein biochemistry and immunoblotting

Cells were washed with ice-cold HBSS, scraped and centrifuged for 10 min at 16,000 *g* at 4 °C. Pellets were resuspended in RIPA buffer (Pierce) complemented with cOmplete protease inhibitor cocktail (Roche) and proteins were extracted on a wheel at 4 °C for at least 30 min. Samples were then centrifuged for 15 min at 16,000 *g* and proteins in the supernatant were processed for protein dosage (BCA, Pierce). SDS–PAGE was performed on samples (15 μg total proteins) loaded in NuPAGE 4–12% Bis-Tris gels (Life technologies). For quantification, wet transfer was performed at 40 V for 135 min at 4 °C (Bio-Rad). For Arf6 deletion confirmation, transfer was performed with iBlot (Life Technologies). Primary and secondary antibodies were incubated overnight at 4 °C and 45 min at room temperature, respectively. Revelation was performed with Immobilon Western Chemiluminescent HRP Substrate (EMD Millipore) and the chemiluminescent signal was imaged with ImageQuant LAS4000 mini (GE Healthcare). Quantification was performed with ImageJ. Uncropped blots are shown in Supplementary Fig. 9.

### Statistical analysis

Statistical calculations were performed using GraphPad Prism software (version 5.02). All the data are given as mean±s.e.m. In most cases, when comparing two samples, two-tailed Student's *t*-test was performed. If a clear hypothesis was postulated that made it valid, then we used a one-tailed *t*-test. When variances were not comparable, Welch's correction was applied. When the distribution could not be assumed to be Gaussian (for compartments size), we used a non parametric Mann–Whitney test. When more samples were compared and Bartlett's test showed that variances could be compared, we used one-way ANOVA or two-way ANOVA with Bonferroni post-tests. If variances could not be compared (*P* value in Bartlett's test<0.05), then we used *t*-tests. Chi-square test was used to compare distributions. Outliers, defined as values that were superior to (mean+3 × s.d.) or inferior to (mean−3 × s.d.) were excluded.

### Data availability

The authors declare that all the data supporting the findings of this study are available within the article and its Supplementary Information files or are available from the corresponding authors on request. *Arf6*^*Flox/+*^ mice generated in this study have been made available to the Jackson Laboratory.

## Additional information

**How to cite this article:** Marquer, C. *et al*. Arf6 controls retromer traffic and intracellular cholesterol distribution via a phosphoinositide-based mechanism. *Nat. Commun.* 7:11919 doi: 10.1038/ncomms11919 (2016).

## Supplementary Material

Supplementary Information Supplementary Figures 1 - 9

Supplementary Movie 1 SIM z-stack of an *Arf6* WT cell immunostained for EEA1 (red) and Vps35 (green), scale bar=5 μm.

Supplementary Movie 2 SIM zoomed-in 3D reconstruction of an *Arf6* WT cell immunostained for EEA1 (red) and Vps35 (green).

Supplementary Movie 3 SIM z-stack of an *Arf6* KO cell immunostained for EEA1 (red) and Vps35 (green), scale bar=5 μm.

Supplementary Movie 4 SIM zoomed-in 3D reconstruction of an *Arf6* KO cell immunostained for EEA1 (red) and Vps35 (green).

Supplementary Movie 5 Spinning-disk confocal live imaging of an *Arf6* WT MEF expressing GFP-SNX6. Scale bar=10 μm.

Supplementary Movie 6 Spinning-disk confocal live imaging of an *Arf6* KO MEF expressing GFP-SNX6. Scale bar=10 μm.

Supplementary Movie 7 Spinning-disk confocal live imaging of an *Arf6* KO cell expressing GFP-SNX6 after 30 minutes of DMSO treatment, scale bar=10 μm.

Supplementary Movie 8 Spinning-disk confocal live imaging of an *Arf6* KO cell before addition of PAO, scale bar=10 μm.

Supplementary Movie 9 Spinning-disk confocal live imaging of the *Arf6* KO cell of movie 8 after 34 minutes of PAO treatment, scale bar=10 μm.
