# Supplementary material for: Arf6 controls retromer traffic and intracellular cholesterol distribution via a phosphoinositide-based mechanism
Source: Nat Commun. 2016 Jun 23;7:11919. doi: 10.1038/ncomms11919 (PMC4931008; doi:10.1038/ncomms11919)
Supplement: Supplementary Information — Supplementary Figures 1 - 9 [file ncomms11919-s1.pdf]

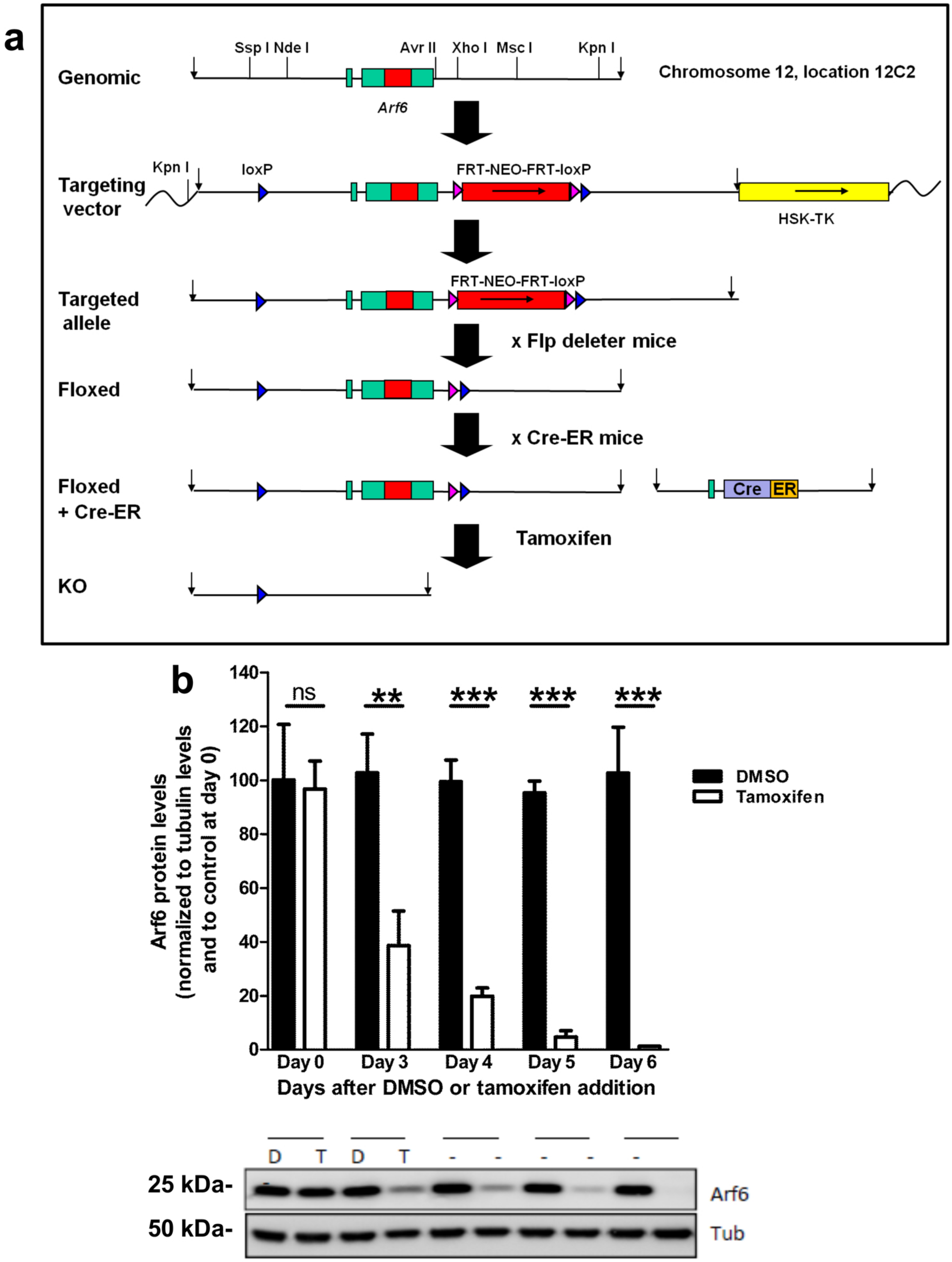

Supplementary Figure 1. ***Arf6* conditional deletion. (a)** Schematic representation of how tamoxifen induces the conditional deletion of *Arf6* in *Arf6$^{Flox/Flox}$; Cre-ER* MEFs. (**b**) Western blot analysis of endogenous Arf6 levels over 6 days in *Arf6$^{Flox/Flox}$; Cre-ER* MEFs treated with vehicle (DMSO, D) or tamoxifen (T) for three days. Tubulin (Tub) was used as an equal loading marker. The bar diagram shows the quantification of Arf6 protein levels, normalised to its levels in cells treated with DMSO at day 0. Values denote mean±SEM (n=3). Ns, ** and *** denote $p>0.05$, $p<0.01$ and $p<0.001$ from a two-way ANOVA with Bonferroni post-tests.

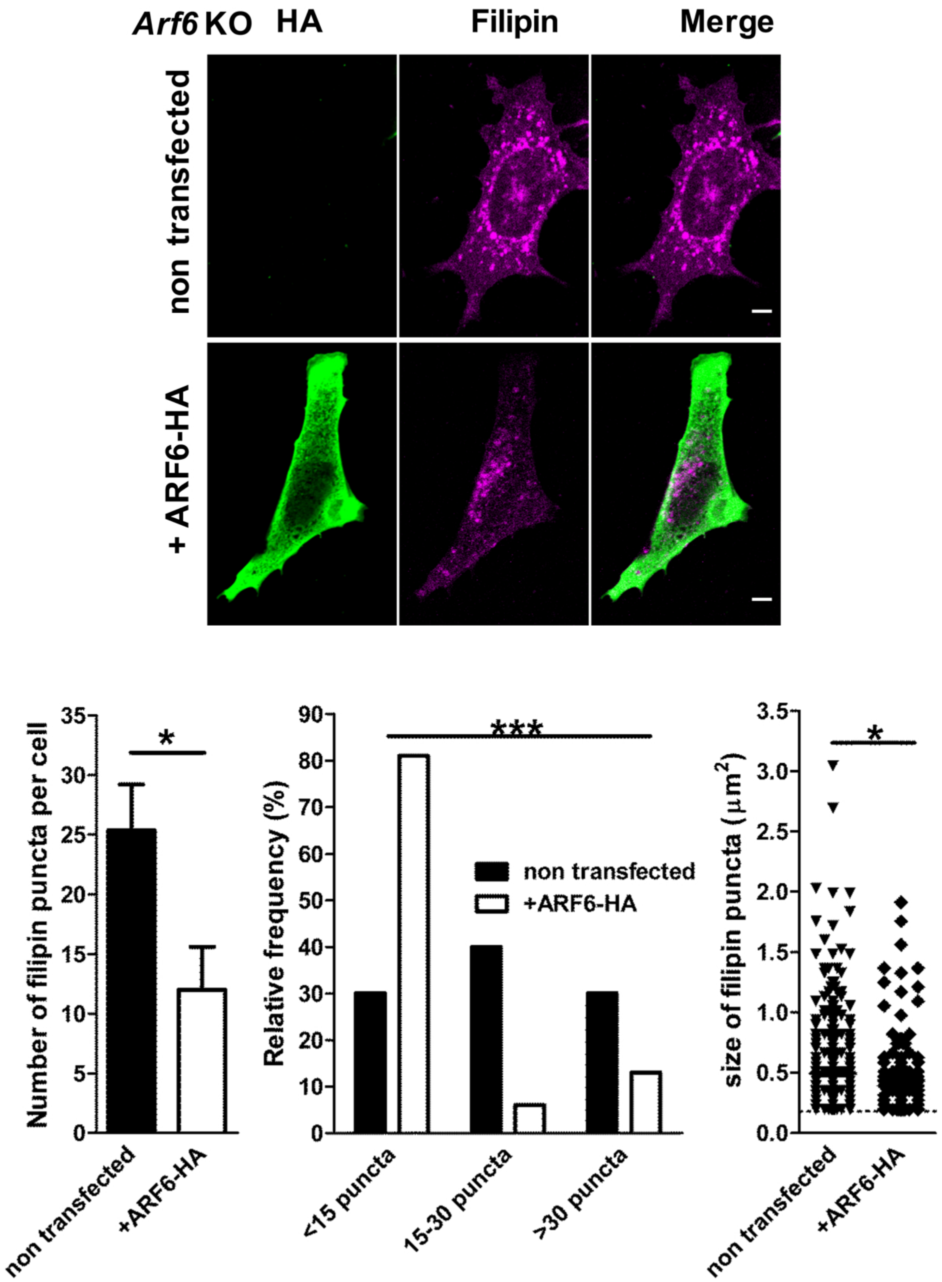

Supplementary Figure 2. Overexpressing ARF6-HA rescues cholesterol redistribution in *Arf6* KO cells. Left, representative confocal images of non-transfected (top) or ARF6-HA overexpressing (green, bottom) *Arf6* KO cells labeled with filipin (magenta). Scale bar=5 μm. Center, quantification of the absolute number and relative distribution of filipin puncta in non-transfected (25±4 puncta/cell, ± indicates SEM, n=20 cells, 2 experiments) or ARF6-HA expressing *Arf6* KO cells (12±4 puncta/cell, n=16 cells, 2 experiments). * denotes $p<0.05$ in Student's t-test. *** denotes $p<0.001$ in chi square test. Right, size of filipin puncta in non-transfected (0.49±0.02 μm^2, ± indicates SEM, 507 puncta, 20 cells) or ARF6-HA expressing *Arf6* KO cells (0.44±0.02 μm^2, 192 puncta, 16 cells). * denotes $p<0.05$ in Mann-Whitney test.

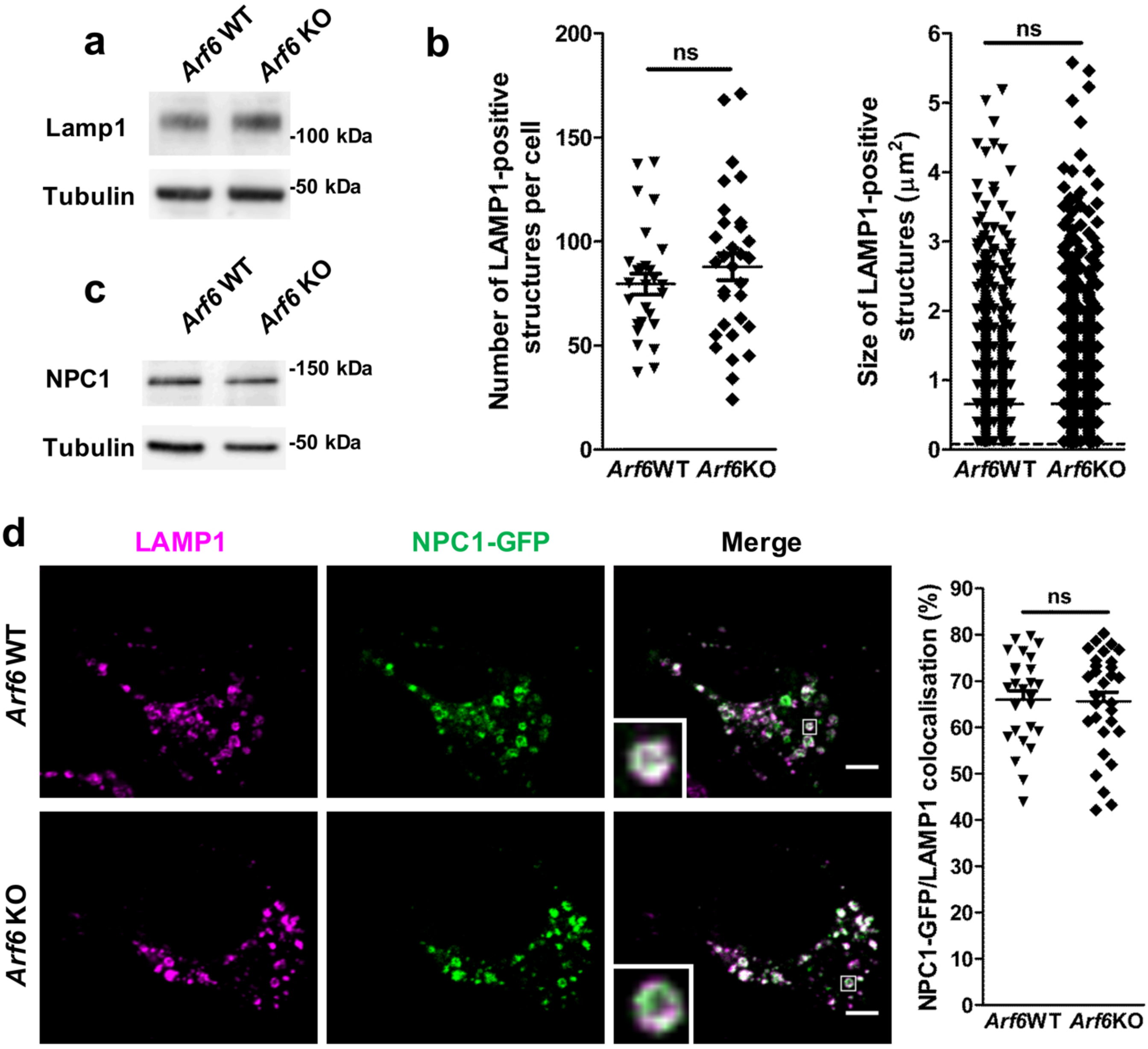

Supplementary Figure 3. The LAMP1 compartment and NPC1-GFP/LAMP1 colocalisation are not affected by Arf6 depletion. (a) Western blot analysis of endogenous LAMP1 levels in *Arf6* WT (100±6 %, ± indicates SEM, n=4) and KO MEFs (137±18 %, n=4), p>0.05 in Student's t-test. Tubulin was used as an equal loading marker. **(b)** Quantification of LAMP1 structures in *Arf6* WT and KO (see confocal images in d). Left, number of LAMP1 structures in WT (80±5 structures/cell, ± indicates SEM, n=28 cells, 3 experiments) and KO cells (88±6 structures/cell, n=31 cells, 3 experiments). ns denotes p>0.05 in Student's t-test. Right, size of LAMP1 structures in WT (0.65±0.01 μm^2, ± indicates SEM, 2228 puncta, 28 cells) and KO cells (0.66±0.01 μm^2, 2724 puncta, 31 cells). ns denotes p>0.05 in Mann-Whitney test. (c) Western blot analysis of endogenous NPC1 levels in *Arf6* WT (100±9 %, ± indicates SEM, n=3) and KO MEFs (75±20 %, n=3), p>0.05 in Student's t-test. Tubulin was used as an equal loading marker. (d) *Arf6* WT and KO cells expressing NPC1-GFP (green) were immunostained for LAMP1 (magenta) and analysed by confocal microscopy. Left, representative images are shown. Scale bar=5 μm. Right, levels of LAMP1/NPC1-GFP colocalisation in *Arf6* WT (66±2 %, ± indicates SEM, n=27 cells, 3 experiments) and KO cells (66±2 %, n= 30 cells, 3 experiments). ns denotes p>0.05 in Student's t-test.

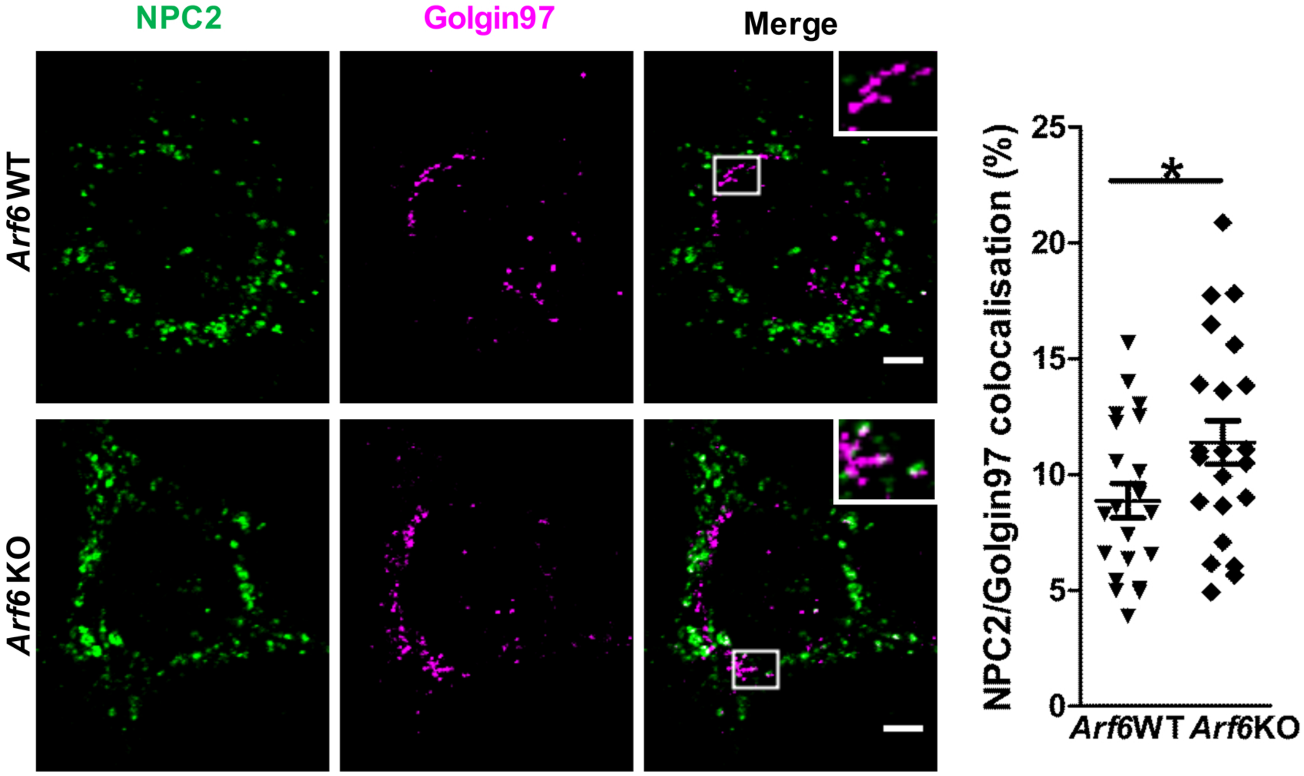

Supplementary Figure 4. NPC2/Golgin97 colocalisation is increased in *Arf6* KO cells. Left panel, representative confocal images of *Arf6* WT and KO cells immunostained for endogenous NPC2 (green) and Golgin97 (magenta). Scale bar=5 µm. Right panel, NPC2/Golgin97 colocalisation levels in *Arf6* WT (8.9±0.7 %, ± indicates SEM, n=21 cells from 2 experiments) and *Arf6* KO cells (11.4±1 %, n=22 cells from 2 experiments). * denotes p<0.05 in Student's t-test.

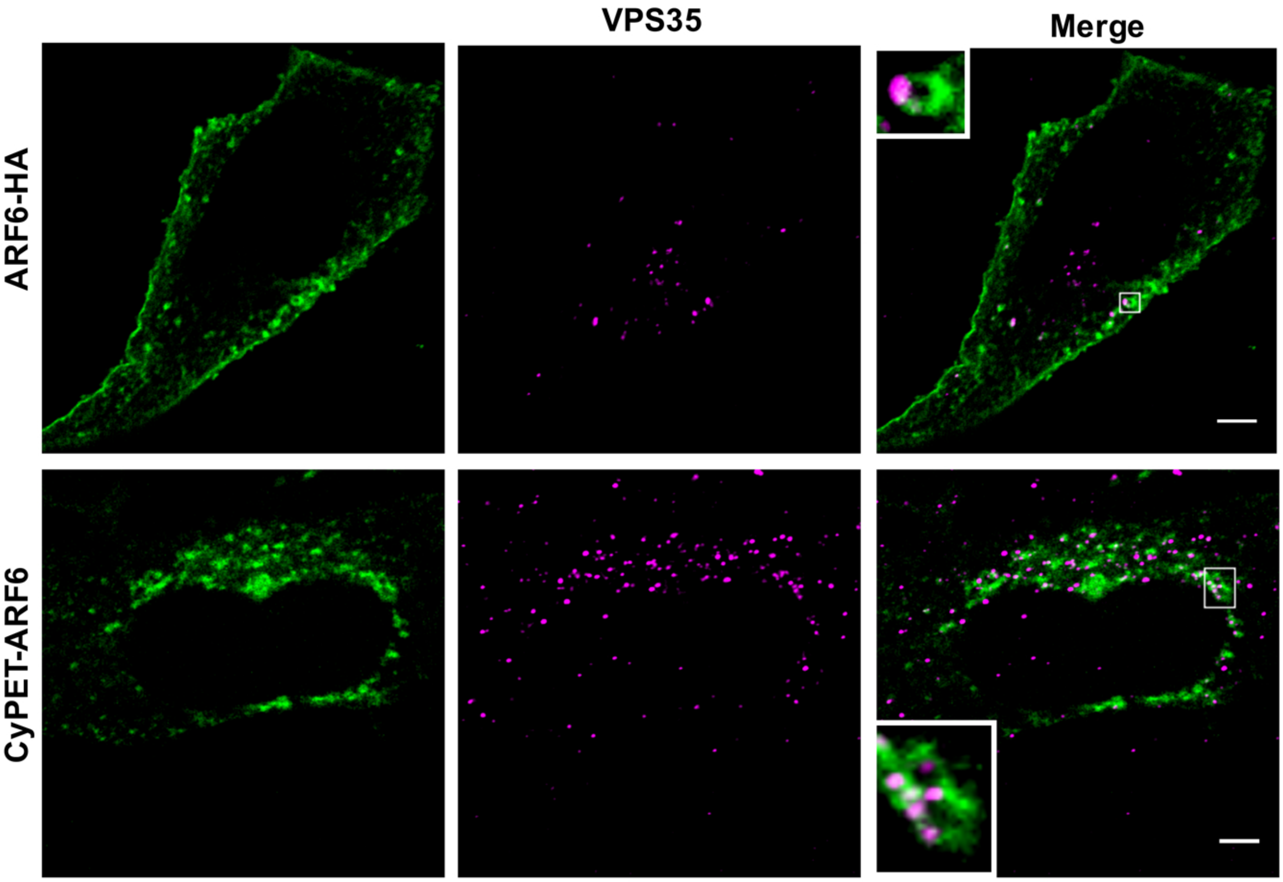

Supplementary Figure 5. A pool of ARF6 and VPS35 is found on the same compartments in HeLa cells. Representative confocal images of HeLa cells overexpressing ARF6-HA (green, top) or CyPET-ARF6 (green, bottom) immunostained for endogenous VPS35 (magenta). Scale bar=5 µm.

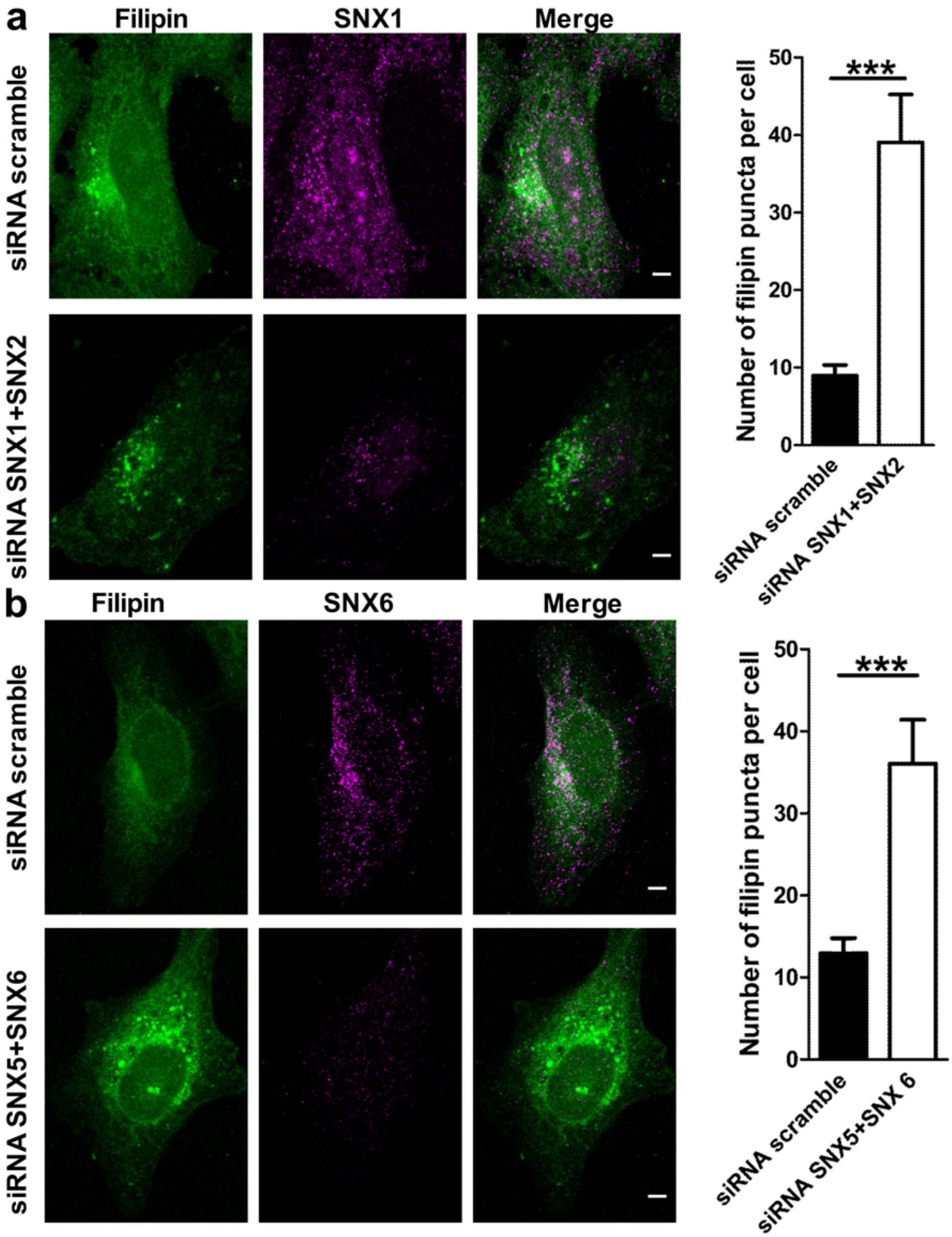

Supplementary Figure 6. Cholesterol is redistributed to cytoplasmic puncta in SNXs KD HeLa cells. (a) Left, representative confocal images of HeLa cells transfected with scramble or (SNX1 and SNX2) siRNAs, immunostained for SNX1 (magenta) and labeled with filipin (green). Scale bar=5 µm. Right, quantification of the number of filipin puncta in scramble (9±1 puncta/cell, ± indicates SEM, n=34 cells, 3 experiments) and (SNX1+SNX2) siRNAs cells (39±6 puncta/cell, n=36 cells, 3 experiments). *** denotes $p<0.001$ in t-test with Welch's correction. **(b)** Left, representative confocal images of HeLa cells transfected with scramble or (SNX5 and SNX6) siRNAs, immunostained for SNX6 (magenta) and labeled with filipin (green). Scale bar=5 µm. Right, quantification of the number of filipin puncta in scramble (13±2 puncta/cell, ± indicates SEM, n=44 cells, 3 experiments) and (SNX5+SNX6) siRNAs cells (36±5 puncta/cell, n=41 cells, 3 experiments). *** denotes $p<0.001$ in t-test with Welch's correction.

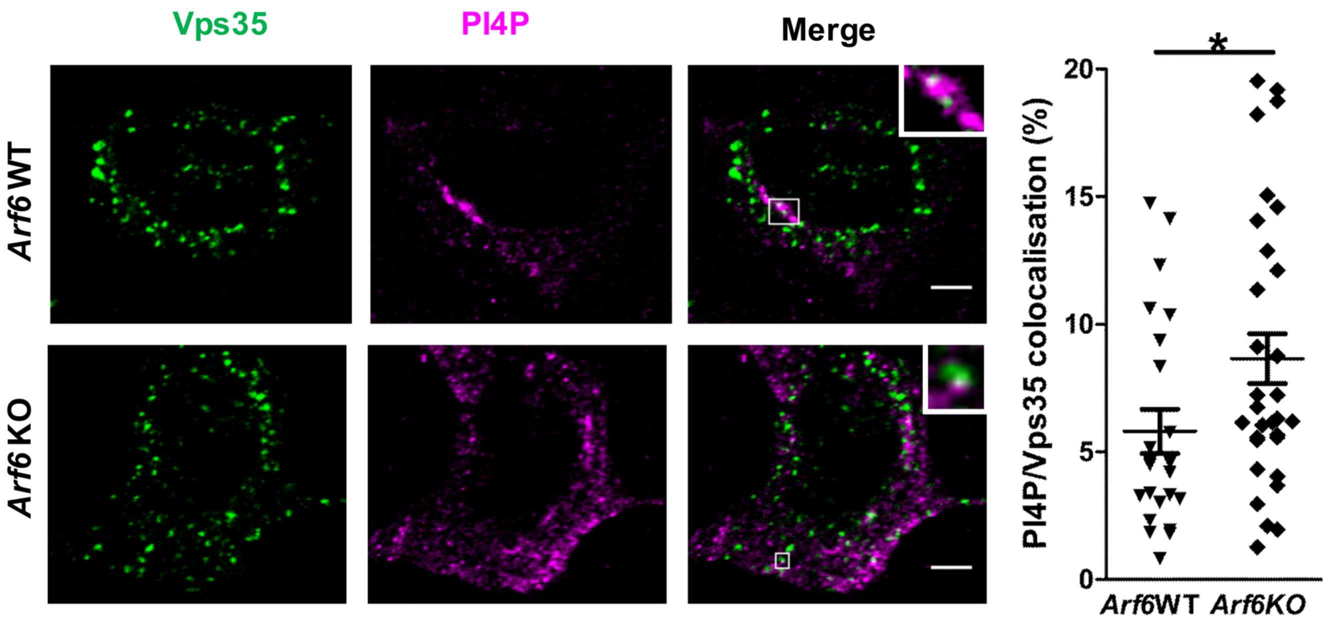

Supplementary Figure 7. PI4P accumulates on Vps35-positive endosomes. Left, representative confocal images of *Arf6* WT and KO cells immunostained for PI4P (magenta) and Vps35 (green). Scale bar=5 µm. Right, PI4P/Vps35 colocalisation levels in WT (5.8±0.9 %, n=23 cells, 3 experiments) and KO cells (8.7±1 %, ± indicates SEM, n=31 cells, 3 experiments). * denotes $p<0.05$ in Student's t-test.

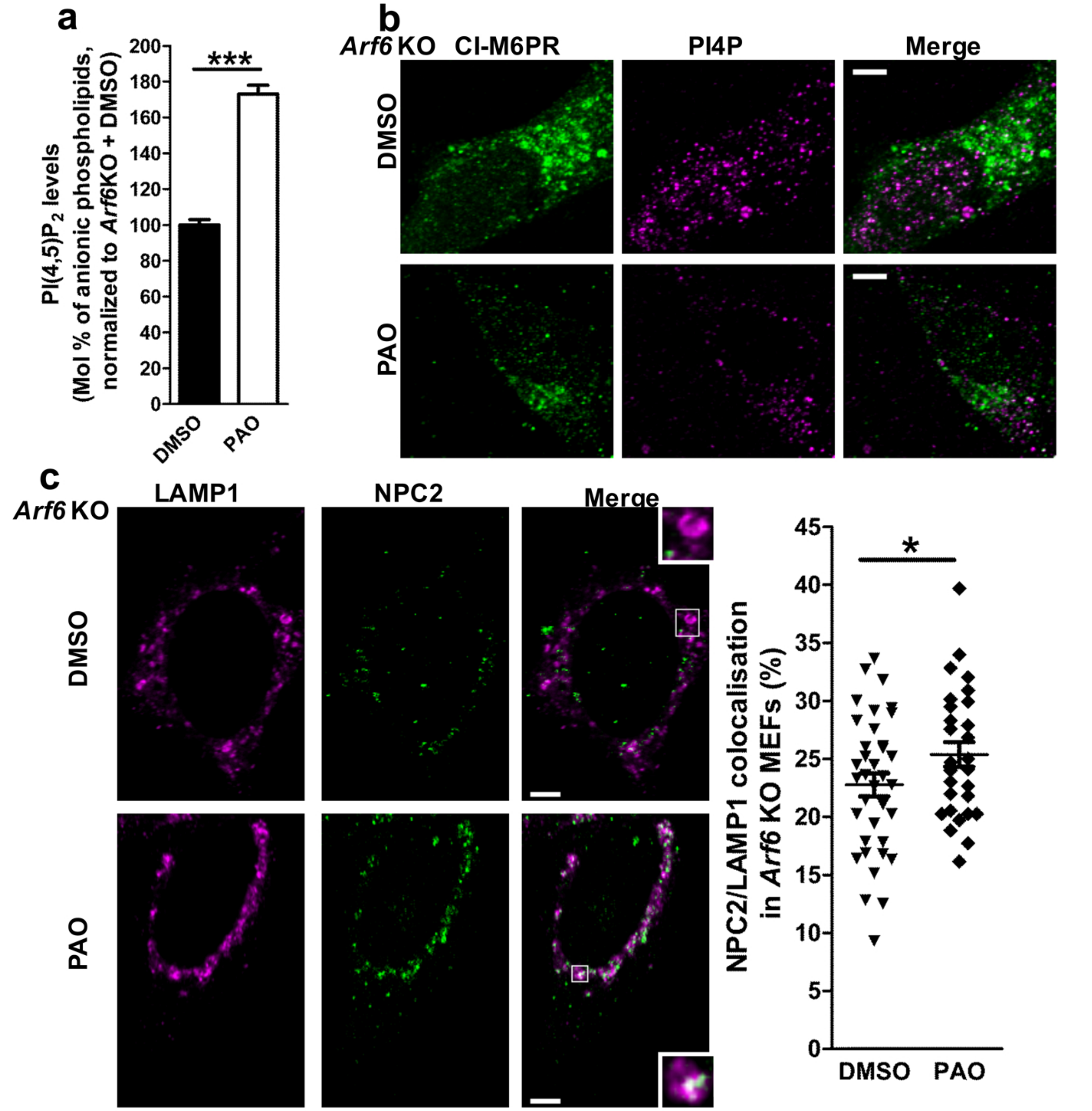

Supplementary Figure 8. PAO treatment normalises CI-M6PR distribution and NPC2/LAMP1 colocalisation in *Arf6* KO cells. (a) Bar diagram showing PI(4,5)P_2 levels in DMSO- and PAO-treated *Arf6* KO cells. Measurements were made by anionic exchange HPLC with suppressed conductivity detection, expressed in molar percentage of total anionic phospholipids measured and normalised to DMSO-treated *Arf6* KO levels. PI(4,5)P_2 levels were increased in PAO-(173±5 %, ± indicates SEM, n=4) compared to DMSO-treated controls (100±3 %, n=4). *** denotes p<0.001 in Student's t-test. **(b)** Representative maximum intensity projections of DMSO- or PAO-treated *Arf6* KO cells immunostained for CI-M6PR (green) and PI4P (magenta). Scale bar=5 µm. **(c)** Left panel, representative confocal images of DMSO- or PAO-treated *Arf6* KO cells immunostained for endogenous LAMP1 (magenta) and NPC2 (green). Scale bar=5 µm. Right panel, NPC2/LAMP1 colocalisation levels in DMSO- (23±1 %, ± indicates SEM, n=36 cells from 3 experiments) and PAO-treated *Arf6* KO cells (25±1 %, n=28 cells from 3 experiments). * denotes p<0.05 in one-tailed Student's t-test.

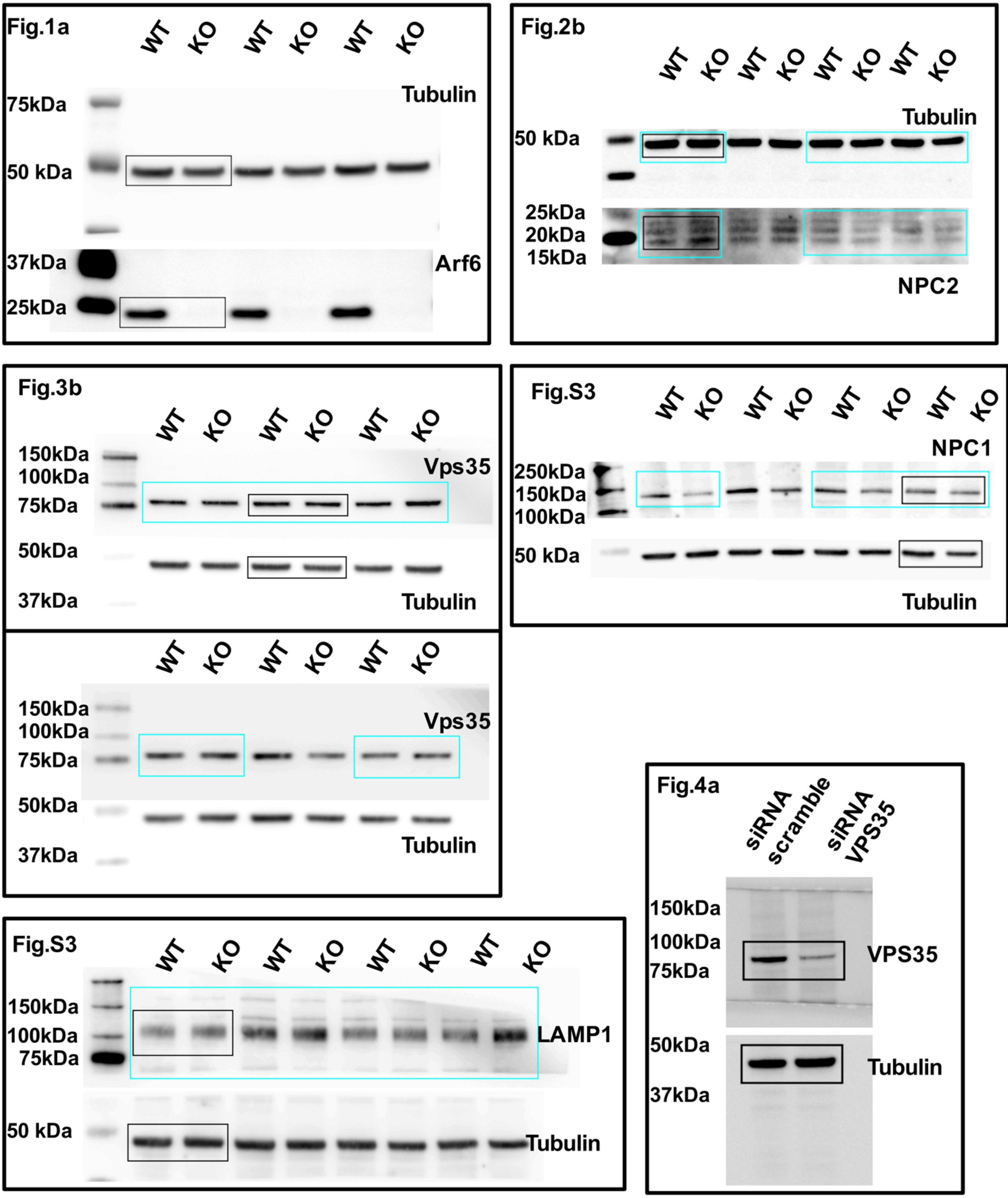

Supplementary Figure 9. Full images of blots that were cropped in main figures. Black squares indicate the cropped images. Blue squares indicate the lines used for quantification.
